# *Bacillus amyloliquefaciens* promotes cluster root formation of white lupin under low phosphorus by mediating auxin levels

**DOI:** 10.1093/plphys/kiae676

**Published:** 2024-12-25

**Authors:** Jinyong Yang, Shenglan Li, Xiangxue Zhou, Chongxuan Du, Ju Fang, Xing Li, Jun Zhao, Fan Ding, Yue Wang, Qian Zhang, Zhengrui Wang, Jianping Liu, Gangqiang Dong, Jianhua Zhang, Feiyun Xu, Weifeng Xu

**Affiliations:** Center for Plant Water-Use and Nutrition Regulation and College of JunCao Science and Ecology, Joint International Research Laboratory of Water and Nutrient in Crop, Fujian Agriculture and Forestry University, Fuzhou 350002, China; Center for Plant Water-Use and Nutrition Regulation and College of JunCao Science and Ecology, Joint International Research Laboratory of Water and Nutrient in Crop, Fujian Agriculture and Forestry University, Fuzhou 350002, China; Center for Plant Water-Use and Nutrition Regulation and College of JunCao Science and Ecology, Joint International Research Laboratory of Water and Nutrient in Crop, Fujian Agriculture and Forestry University, Fuzhou 350002, China; Center for Plant Water-Use and Nutrition Regulation and College of JunCao Science and Ecology, Joint International Research Laboratory of Water and Nutrient in Crop, Fujian Agriculture and Forestry University, Fuzhou 350002, China; Center for Plant Water-Use and Nutrition Regulation and College of JunCao Science and Ecology, Joint International Research Laboratory of Water and Nutrient in Crop, Fujian Agriculture and Forestry University, Fuzhou 350002, China; Center for Plant Water-Use and Nutrition Regulation and College of JunCao Science and Ecology, Joint International Research Laboratory of Water and Nutrient in Crop, Fujian Agriculture and Forestry University, Fuzhou 350002, China; Center for Plant Water-Use and Nutrition Regulation and College of JunCao Science and Ecology, Joint International Research Laboratory of Water and Nutrient in Crop, Fujian Agriculture and Forestry University, Fuzhou 350002, China; Center for Plant Water-Use and Nutrition Regulation and College of JunCao Science and Ecology, Joint International Research Laboratory of Water and Nutrient in Crop, Fujian Agriculture and Forestry University, Fuzhou 350002, China; Center for Plant Water-Use and Nutrition Regulation and College of JunCao Science and Ecology, Joint International Research Laboratory of Water and Nutrient in Crop, Fujian Agriculture and Forestry University, Fuzhou 350002, China; Center for Plant Water-Use and Nutrition Regulation and College of JunCao Science and Ecology, Joint International Research Laboratory of Water and Nutrient in Crop, Fujian Agriculture and Forestry University, Fuzhou 350002, China; Center for Plant Water-Use and Nutrition Regulation and College of JunCao Science and Ecology, Joint International Research Laboratory of Water and Nutrient in Crop, Fujian Agriculture and Forestry University, Fuzhou 350002, China; Center for Plant Water-Use and Nutrition Regulation and College of JunCao Science and Ecology, Joint International Research Laboratory of Water and Nutrient in Crop, Fujian Agriculture and Forestry University, Fuzhou 350002, China; Amway (China) Botanical R&D Centre, Wuxi 214145, China; State Key Laboratory of Agrobiotechnology, The Chinese University of Hong Kong, Shatin, Hong Kong 999077, China; Department of Biology, Hong Kong Baptist University, Hong Kong 999077, China; Center for Plant Water-Use and Nutrition Regulation and College of JunCao Science and Ecology, Joint International Research Laboratory of Water and Nutrient in Crop, Fujian Agriculture and Forestry University, Fuzhou 350002, China; Center for Plant Water-Use and Nutrition Regulation and College of JunCao Science and Ecology, Joint International Research Laboratory of Water and Nutrient in Crop, Fujian Agriculture and Forestry University, Fuzhou 350002, China

## Abstract

White lupin (*Lupinus albus* L.) produces cluster roots to acquire more phosphorus under phosphorus deficiency. *Bacillus amyloliquefaciens* SQR9 contributes to plant growth, but whether and how it promotes cluster root formation in white lupin remain unclear. Here, we investigated the roles of SQR9 in cluster root formation under low phosphorus conditions using a microbial mutant and virus-induced gene silencing (VIGS) in white lupin. SQR9 substantially enhanced cluster root formation under low phosphorus conditions. The *ysnE* gene encodes an auxin biosynthesis enzyme in SQR9 and was associated with cluster root formation, as *ysnE*-defective SQR9 did not trigger cluster root formation. SQR9 inoculation induced the expression of PIN-formed2 (*LaPIN2*, encoding an auxin transporter) and YUCCA4 (*LaYUC4*, encoding an auxin biosynthesis enzyme) in white lupin roots. VIGS-mediated knockdown of *LaPIN2* and *LaYUC4* prevented wild-type SQR9-induced cluster root formation in white lupin. Finally, white lupin *LaYUC4*-derived auxin and SQR9-derived auxin pools were both transported by LaPIN2, promoting cluster root formation under low phosphorus conditions. Taken together, we propose that *B. amyloliquefaciens* promotes cluster root formation in white lupin under low phosphorus conditions by stimulating auxin biosynthesis and transport. Our results provide insights into the interplay between bacteria and root auxin in crop phosphorus use efficiency.

## Teaser

*Bacillus amyloliquefaciens* produces auxin and promotes auxin biosynthesis in white lupin under low phosphorus conditions, triggering cluster root formation.

## Introduction

Phosphorus is an essential element for plants (López-Arredondo et al. 2014; Liu 2021) that plays a crucial role in respiration, photosynthesis, and the regulation of various enzymes (Rubio et al. 2001; Zhu et al. 2016; Lambers 2022). Deficient of available phosphorus can significantly hinder plant growth and development (Rouached et al. 2011; Liu et al. 2024). However, more than 80% of applied phosphorus can become immobile and unavailable for uptake by plants due to factors such as precipitation with calcium, iron oxides/hydroxides, or adsorption by aluminum, as well as conversion to organic forms in the soil (Richardson and Simpson 2011; Xu et al. 2012; Li et al. 2014). To address these challenges, plants have evolved a range of strategies to increase phosphorus acquisition from the soil. Roots can modify their morphological structure and physiological function in response to low phosphorus levels, including developing symbiotic associations with soil microorganisms (Lidbury et al. 2020), modifying their architecture and branching (Marschner et al. 1986), increasing root hair density and length (Reid 1981; Miguel et al. 2015), exuding various compounds (Duff et al. 1994; Xu et al. 2020), and developing cluster roots (Rourke et al. 2013; Raven et al. 2018).

White lupin (*Lupinus albus* L.) can develop cluster roots with high phosphorus use efficiency in soils with low phosphorus levels (Yan et al. 2002; Hufnagel et al. 2021). Such roots are closely arranged along the primary or secondary lateral roots, with more than 15 short lateral rootlets per centimeter, and undergo limited growth (generally ≤2 cm) ([Bibr kiae676-B39][Bibr kiae676-B39]; Olt et al. 2024). They secrete large amounts of organic acids, phosphatases, and protons, acidifying the surrounding rhizosphere and allowing plants to take up phosphorus from the soil (Wang et al. 2010; Cheng et al. 2014). Previous studies suggest that C-terminally encoded peptide receptor 1 (*CEP1*) and ATP-binding cassette transporter 37 (*ABCG37*) might play a role in cluster root formation of white lupin under low phosphorus conditions (Zhou et al. 2019; Xu et al. 2020). In addition, microorganisms are also involved in cluster root formation of white lupin. For example, the soil-borne bacterium *Klebsiella pneumoniae* has been shown to promote white lupin cluster root formation under low phosphorus via ethylene signaling (Zhang et al. 2023).

The microbiome of the rhizosphere is referred to as the second genome of plants (Berendsen et al. 2012; Zhang et al. 2022b). Interactions between roots and rhizosphere microorganisms are important for enhancing plant growth and yield (Talboys et al. 2014; Liu et al. 2016, 2023). Various bacteria isolated from the rhizosphere or endosphere of plants have been shown to possess plant growth–promoting activities. For instance, some Bacillus species are plant growth–promoting rhizosphere (PGPR) bacteria that significantly enhance plant growth (Erturk et al. 2010; Hossain et al. 2019; Tzipilevich et al. 2021). *Bacillus amyloliquefaciens* SQR9, isolated from the rhizosphere of cucumbers, is a PGPR that exhibits strong root colonization ability (Cao et al. 2011; Qiu et al. 2012). It produces the antifungal compound bacillomycin D, which is involved in biofilm formation and root colonization (Xu et al. 2013). In addition, it produces auxin and promotes the growth and development of lateral roots in Arabidopsis (*Arabidopsis thaliana*) (Shao et al. 2015; Li et al. 2021). However, it is unclear whether it can promote cluster root formation in white lupin.

In this study, we explored the effects of *B. amyloliquefaciens* SQR9 on cluster root formation in white lupin. Then, molecular genetics and pharmacological approaches were used to further investigate the role of auxin in regulating cluster root formation. Our study provided a tentative model to elucidate the mechanisms by which *B. amyloliquefaciens* SQR9 influences cluster root formation in white lupin.

## Results

### *B. amyloliquefaciens* SQR9 increased the cluster root formation of white lupin under low phosphorus conditions

**Figure 1.**
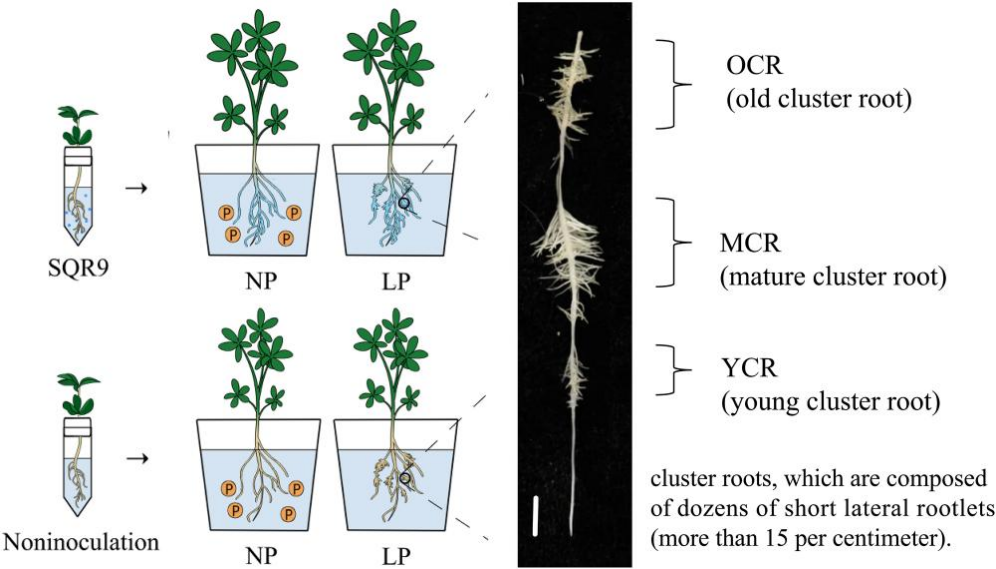
Experimental setup for inoculating white lupin with *B. amyloliquefaciens* SQR9 and cluster root phenotype (with Supplementary Fig. S1). Phenotypes of 3 developmental stages of cluster roots (from young to old) were formed under LP conditions. Cluster roots are composed of dozens of short lateral rootlets (more than 15/cm). Under NP supply (the circle P represents phosphates), white lupin has no cluster roots (bar = 1 cm). NP, normal phosphorus; LP, low phosphorus.

**Figure 2.**
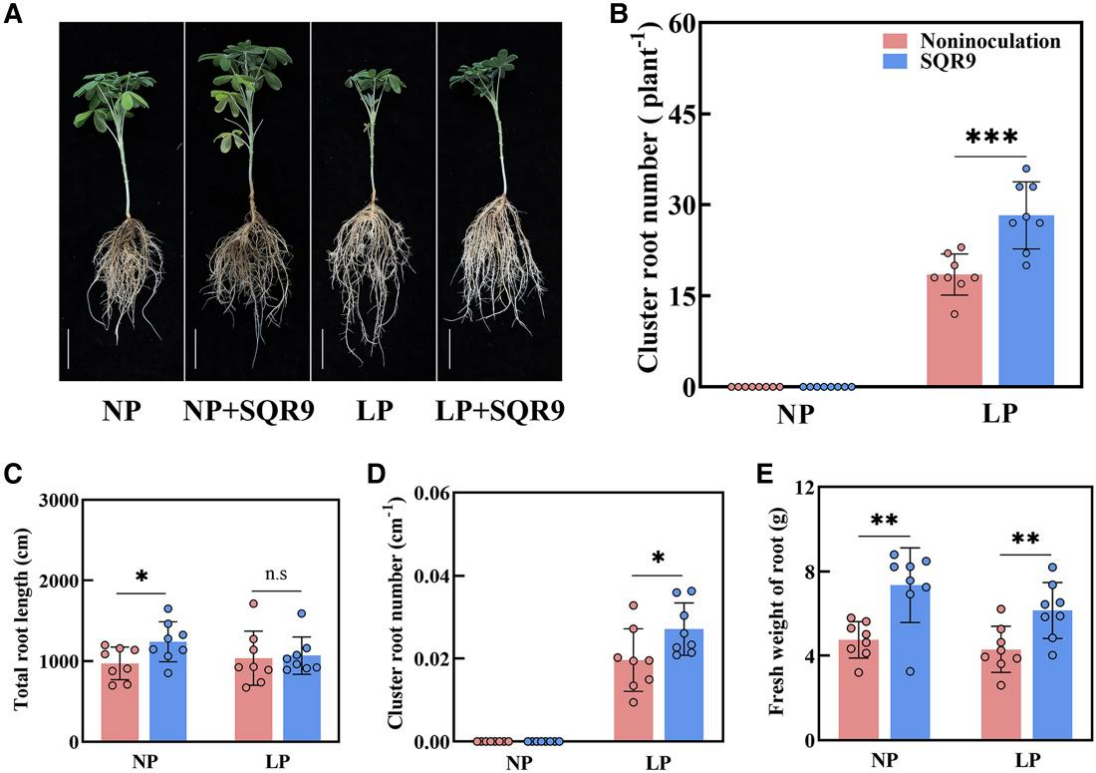
*B. amyloliquefaciens* SQR9 promotes cluster root formation of white lupin under low phosphorus conditions. **A)** Phenotypes of white lupin with or without SQR9 inoculation under NP and LP conditions (bars = 5 cm). **B** to **E)** Cluster root number, total root length, cluster root number per centimeter (cm) of total root, and root fresh weight of white lupin with or without SQR9 inoculation under NP and LP conditions. Data are means ± Sds (*n* = 8). In all panels, the significant differences were determined using 2-sided Student's *t*-test. *P < 0.05, **P < 0.01, ***P < 0.001. Statistics of cluster root number include all developmental stages. Noninoculation, no *B. amyloliquefaciens* inoculation; SQR9, *B. amyloliquefaciens* inoculation; NP, normal phosphorus; LP, low phosphorus; n.s, nonsignificant.

To evaluate the effects of *B. amyloliquefaciens* SQR9 on white lupin growth, the SQR9-inoculated or noninoculated plants were cultivated under normal phosphorus or low phosphorus conditions (Fig. 1). Fluorescence of the marker GFP was observed in white lupin roots after inoculation with GFP-labeled SQR9 (SQR9-GFP) (Supplementary Fig. S1). White lupin did not form cluster roots, neither SQR9-inoculated nor noninoculated plants, under normal phosphorus supply conditions (Fig. 2A); thus, we focused on the role of SQR9 in cluster root formation under low phosphorus conditions. The number of cluster roots inoculated with SQR9 was significantly higher than that in SQR9-noninoculated plants under low phosphorus conditions (Fig. 2B). Additionally, total root length of plants inoculated with SQR9 was significantly increased compared with noninoculated plants under normal phosphorus. However, there was no significant difference in total root length under low phosphorus conditions (Fig. 2C). Furthermore, the cluster root number per centimeter (cm) of the total roots in SQR9-inoculated plants was significantly higher than that in noninoculated plants under low phosphorus conditions (Fig. 2D). In addition, root fresh weight with SQR9 inoculation was significantly increased compared with noninoculated plants under normal and low phosphorus conditions, respectively (Fig. 2E). Taken together, these results showed that SQR9 inoculation promoted cluster root formation of white lupin under low phosphorus conditions.

### Auxin produced from *B. amyloliquefaciens* SQR9 by its *ysnE* gene was involved in cluster root formation of white lupin under low phosphorus

**Figure 3.**
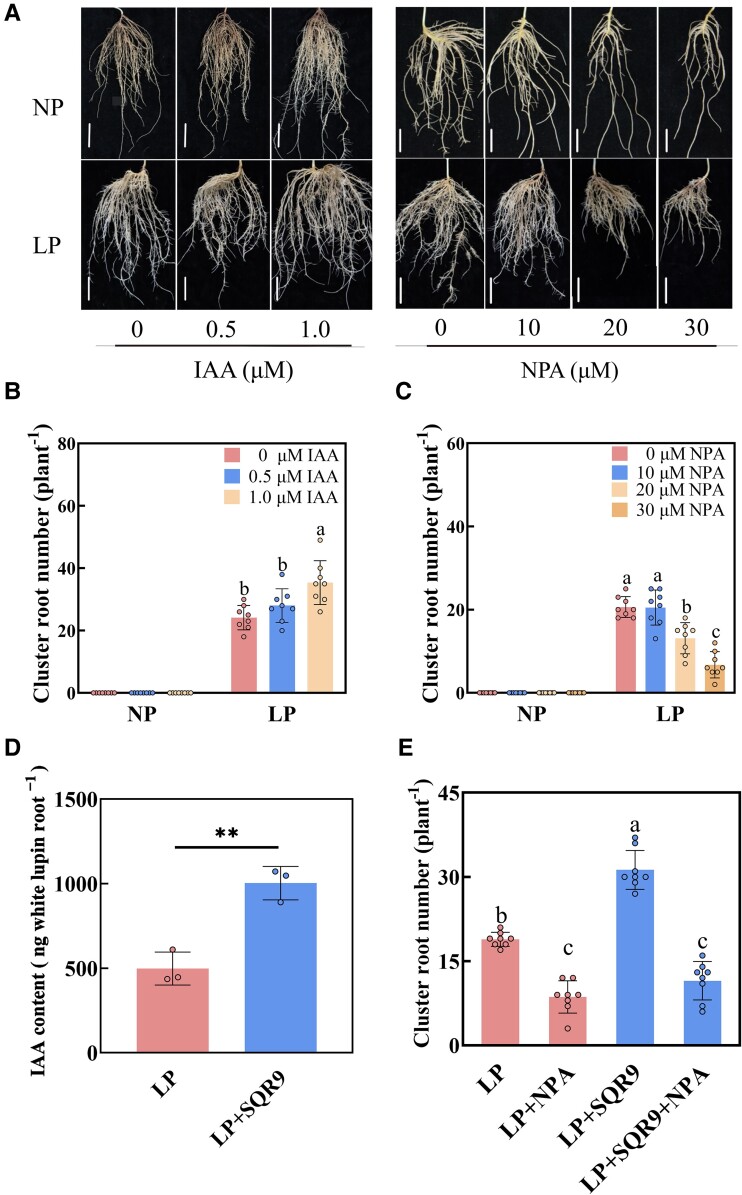
Auxin is involved in the cluster root formation of white lupin inoculated with *B. amyloliquefaciens* SQR9 under LP conditions (with Supplementary Figs. S2 to S4). **A)** Phenotypes of roots in white lupin with IAA (0, 0.5, and 1.0 *μ*m) or NPA (0, 10, 20, and 30 *μ*m) under NP and LP conditions (bars = 5 cm). **B)** Cluster root number of white lupin with 0, 0.5, and 1.0 *μ*m IAA under NP and LP conditions. Data are means ± Sds (*n* = 8). Bars with different letters indicate significant differences among treatments (*P* < 0.05, ANOVA, Duncan's multiple range test). **C)** Cluster root number of white lupin with 0, 10, 20, and 30 *μ*m NPA under NP and LP conditions. Data are means ± Sds (*n* = 8). Bars with different letters indicate significant differences among treatments (*P* < 0.05, ANOVA, Duncan's multiple range test). **D)** IAA content of roots in white lupin with or without SQR9 inoculation under LP conditions. Data are means ± Sds (*n* = 3). Asterisks indicate significant differences among treatments (**P < 0.01; 2-sided Student's *t*-test). **E)** Cluster root number of white lupin without inoculation under LP conditions, treated with 30 *μ*m NPA under LP conditions (LP + NPA), inoculated with SQR9 under LP conditions (LP + SQR9), and inoculated with SQR9 under LP conditions treated with 30 *μ*m NPA (LP + SQR9 + NPA). Data are means ± Sds (*n* = 8). Bars with different letters indicate significant differences among treatments (*P* < 0.05, ANOVA, Duncan's multiple range test). SQR9, inoculated with *B. amyloliquefaciens* SQR9; NP, normal phosphorus; LP, low phosphorus.

**Figure 4.**
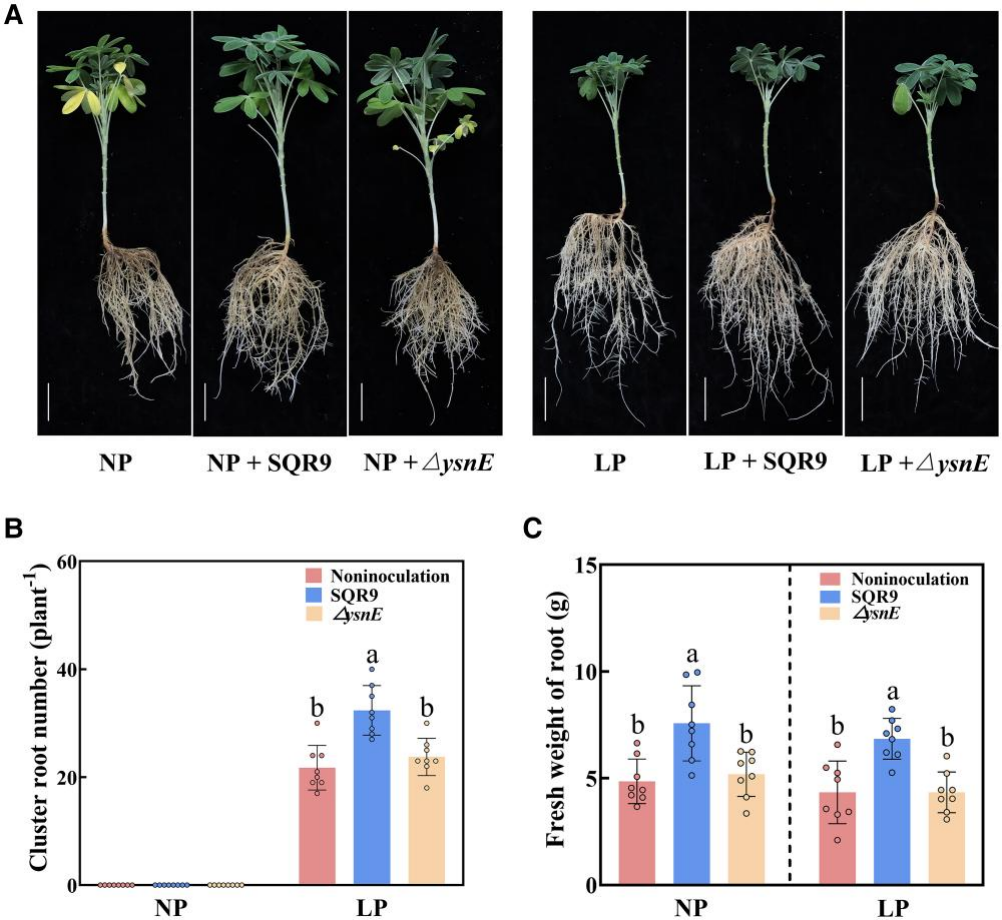
The auxin synthesis gene *ysnE* of *B. amyloliquefaciens* SQR9 is important for SQR9-induced cluster root formation under LP conditions (with Supplementary Fig. S5). **A)** Phenotypes of white lupin without inoculation under NP and LP conditions, inoculated with SQR9 under NP (NP + SQR9) and LP conditions (LP + SQR9) and inoculated with *ΔysnE* mutant under NP (NP + *ΔysnE*) and LP conditions (LP + *ΔysnE*). Bars = 5 cm. **B** to **C)**, Cluster root number and root fresh weight of white lupin without inoculation, inoculated with SQR9 and inoculated with *ΔysnE* mutant under NP and LP conditions. Data are means ± Sds (*n* = 8). In all panels, bars with different letters indicate significant differences among treatments (*P* < 0.05, ANOVA, Duncan's multiple range test). NP, normal phosphorus; LP, low phosphorus; SQR9, *B. amyloliquefaciens* SQR9; *ΔysnE*, *ysnE*-deficient mutant in the SQR9 genetic background.

To explore the relation between auxin and cluster root formation in white lupin, indole-3-acetic acid (IAA, a type of auxin) and the auxin transport inhibitor 1-naphthylphthalamic acid (NPA) were applied. At an IAA concentration of 0.5 *μ*m, the cluster root number increased by 16.1% relative to untreated plants under low phosphorus conditions. At an IAA concentration of 1.0 *μ*m, it significantly increased by 46.6% (Fig. 3, A and B). At an NPA concentration of 30 *μ*m, the cluster root number was significantly decreased by 67.7% compared with the untreated plants under low phosphorus conditions (Fig. 3, A and C). In addition, the auxin precursor L-tryptophan and the auxin biosynthesis inhibitor L-kynurenine were used to verify the role of auxin in white lupin cluster root formation. At an L-tryptophan concentration of 50 *μ*m, the cluster root number increased by 17.6% relative to untreated plants under low phosphorus conditions. At an L-tryptophan concentration of 200 *μ*m, it significantly increased by 41.8% (Supplementary Fig. S2A). At an L-kynurenine concentration of 10 *μ*m, the cluster root number was significantly decreased by 28.0% compared with the untreated plants under low phosphorus conditions. At an L-kynurenine concentration of 30 *μ*m, the cluster root number was significantly decreased by 77.2% compared with the untreated plants under low phosphorus conditions (Supplementary Fig. S2B). The plants with the addition of L-tryptophan increased cluster root formation, while L-kynurenine showed the opposite effect (Supplementary Fig. S2, A and B). Furthermore, the IAA content of the SQR9-inoculated white lupin was significantly increased compared with the noninoculated plants (Fig. 3D). Fluorescence intensity of DR5rev::GFP in SQR9-inoculated white lupin was also significantly increased compared with the noninoculated plants (Supplementary Fig. S3, A to C). Next, compared with noninoculated plants, the cluster root number of noninoculated plants with the addition of 30 *μ*m NPA was significantly decreased. Compared with SQR9-inoculated plants, the cluster root number of SQR9-inoculated plants with the addition of 30 *μ*m NPA was significantly reduced (Fig. 3E). We also examined the sucrose levels in white lupin root and found no difference between SQR9-inoculated and noninoculated plants both under normal phosphorus and low phosphorus (Supplementary Fig. S4), indicating that sucrose was not possibly involved in SQR9-induced plant growth of white lupin. To further verify the role of SQR9-produced auxin in white lupin cluster root formaiton, *ysnE* (a key gene for auxin biosynthesis in SQR9)–knockout mutant (*ΔysnE*) in the SQR9 background was used (Fig. 4A). The *ΔysnE* showed the 73.7% lower IAA content than SQR9 (Supplementary Fig. S5). The cluster root number of white lupin inoculated with *ΔysnE* was significantly reduced in comparison with SQR9-inoculated plants (Fig. 4B). In addition, the root fresh weight of *ΔysnE-*inoculated plants was significantly reduced under conditions of normal and low phosphorus, respectively, compared with SQR9-inoculated plants (Fig. 4C). Taken together, these results showed that the SQR9-produced auxin by its *ysnE* gene was necessary for SQR9-promoting cluster root formation in white lupin.

### White lupin *LaYUC4* and *LaPIN2* genes were required for cluster root formation under low phosphorus conditions with *B. amyloliquefaciens* SQR9 inoculation

**Figure 5.**
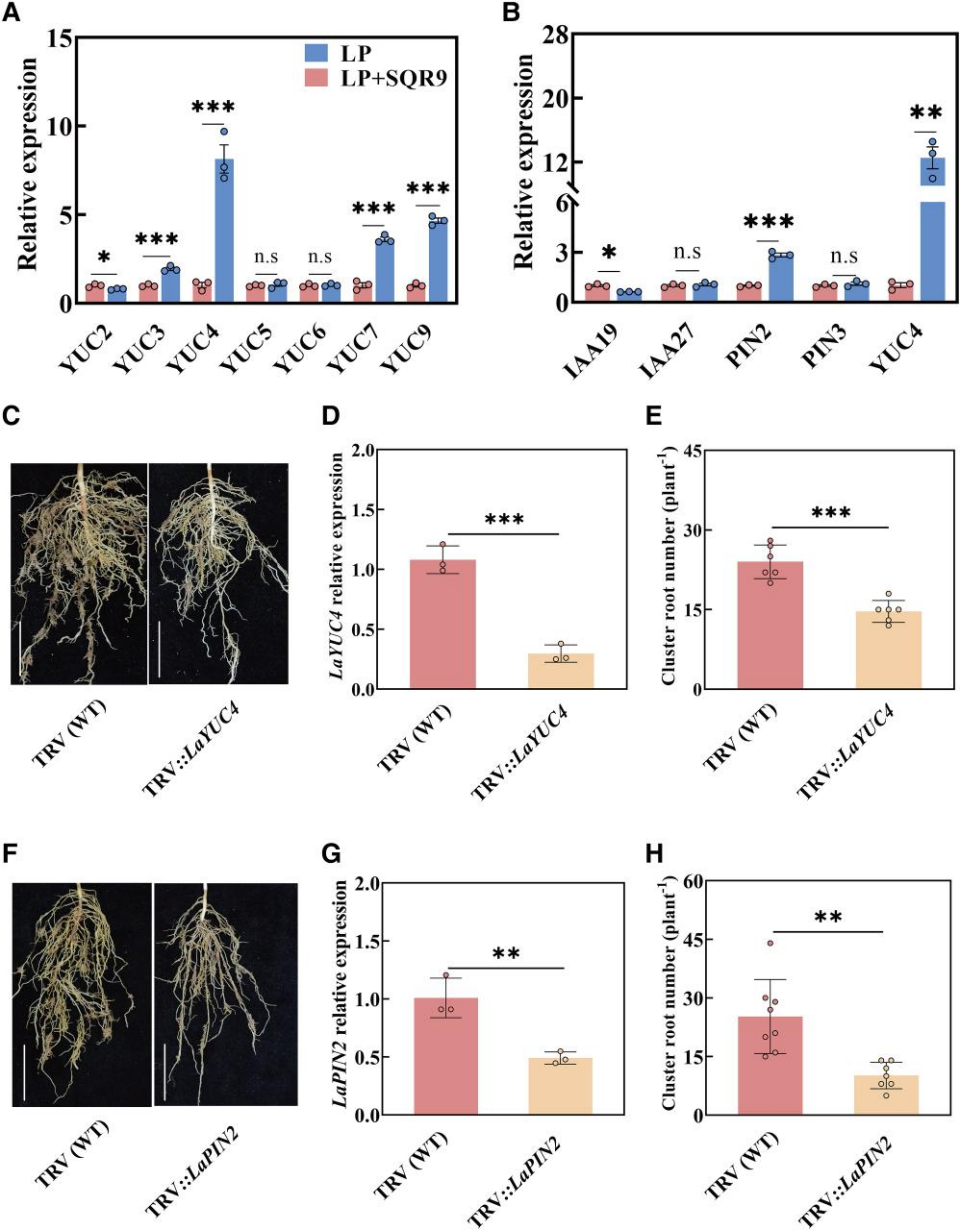
White lupin auxin synthesis gene *LaYUC4* or auxin transport gene *LaPIN2* was mediated by SQR9 inoculation under low phosphorus and construction of VIGS lines for the 2 genes (with Supplementary Fig. S6). **A** and **B)** Expression levels of auxin-related genes in white lupin roots without inoculation (LP) or inoculated with SQR9 (LP + SQR9) under low phosphorus conditions. Data are means ± Sds (*n* = 3). **C)** Phenotypes of roots of white lupin *LaYUC4*-silenced (TRV::*LaYUC4*) and empty vector control, TRV (WT). Bars = 5 cm. **D)** Expression levels of *LaYUC4* in TRV::*LaYUC4* and TRV (WT). Data are means ± Sds (*n* = 3). **E)** Cluster root number of white lupin with TRV::*LaYUC4* and TRV (WT) under low phosphorus conditions. Data are means ± Sds (*n* = 6). **F)** Phenotypes of roots in white lupin *LaPIN2*-silenced (TRV::*LaPIN2*) and empty vector control, TRV (WT). Bars = 5 cm. **G)** Expression levels of *LaPIN2* in TRV::*LaPIN2* and TRV (WT). Data are means ± Sds (*n* = 3). **H)** Cluster root number of white lupin with TRV::*LaPIN2* and TRV (WT) under low phosphorus conditions. Data are means ± Sds (*n* = 7–8). In all panels, the significant differences were determined using 2-sided Student's *t*-test; n.s, nonsignificant; *P < 0.05, **P < 0.01, ***P < 0.001.

We hypothesized that SQR9 may enhance cluster root formation by stimulating the white lupin auxin synthesis. Then, we determined auxin-related gene expression levels using RNA-seq data. The *YUCCA* (*LaYUC*) gene family is important for auxin biosynthesis in white lupin. *LaYUC4*, *LaYUC7*, and *LaYUC9* of cluster roots were significantly upregulated compared with lateral roots without cluster roots (Supplementary Fig. S6). To further examine which YUC gene played the key role in SQR9-induced cluster root formation, we analyzed the relative expression of YUC genes in SQR9-inoculated plants under low phosphorus conditions. The expression of *LaYUC4* was upregulated in SQR9-inoculated plants compared with noninoculated plants under low phosphorus conditions (Fig. 5A). The relative expression of *LaYUC4* was the highest in *LaYUC4*, *LaYUC7*, and *LaYUC9*; thus, *LaYUC4* may play an important role in SQR9-induced cluster root formation. Then, plants with *LaYUC4* knockdown were generated by virus-induced gene silencing (VIGS). *LaYUC4* gene expression of *LaYUC4* knockdown plants (TRV::*LaYUC4*) was significantly reduced compared with control plants, TRV (WT), carrying the empty vector alone under low phosphorus conditions. Cluster root number of *LaYUC4* knockdown plants (TRV::*LaYUC4*) was significantly decreased compared with TRV (WT) under low phosphorus conditions (Fig. 5, C to E).

The *LaPIN* gene family is important for auxin transport in white lupin. Furthermore, *LaPIN2* of SQR9-inoculated plants was significantly upregulated compared with noninoculated roots under low phosphorus conditions (Fig. 5B). Plants with *LaPIN2* knockdown were generated by VIGS. *LaPIN2* knockdown plants (TRV::*LaPIN2*) showed a significant reduction of *LaPIN2* gene expression (Fig. 5, F and G). TRV::*LaPIN2* showed a significantly decrease in cluster root number under low phosphorus conditions compared with TRV (WT) (Fig. 5H).

**Figure 6.**
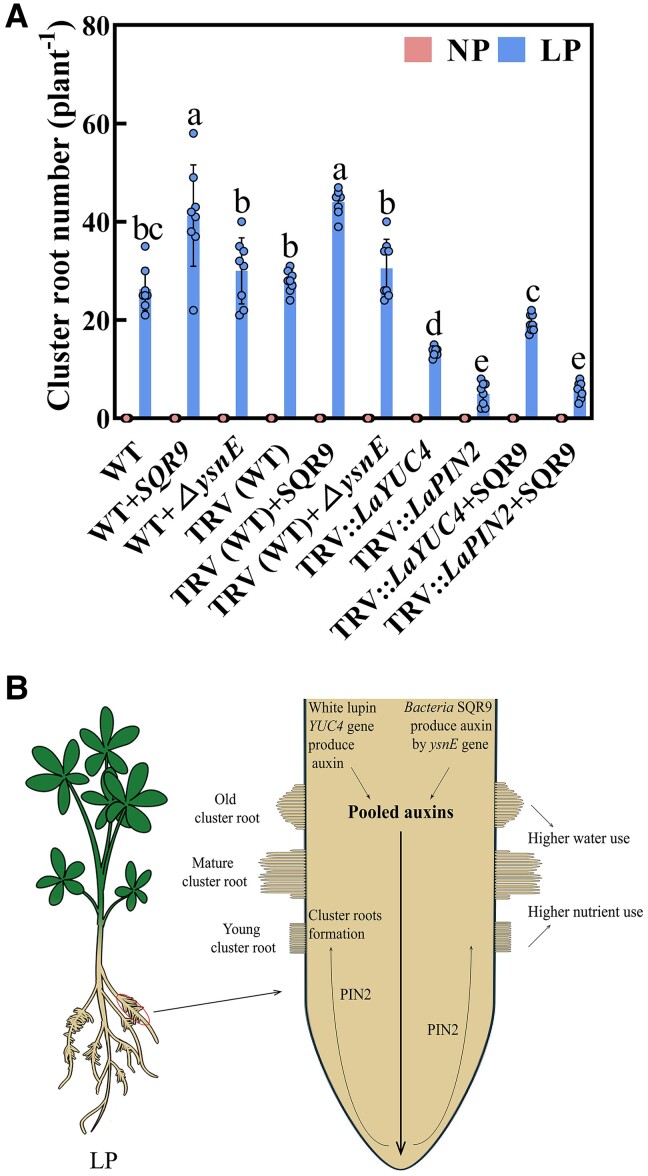
*B. amyloliquefaciens* promotes the cluster root formation of white lupin under LP through auxins mediation (auxin biosynthesis and transport). **A)** Cluster root number of white lupin with different treatments under NP and LP conditions. Data are means ± Sds (*n* = 8). Bars with different letters indicate significant differences among treatments (*P* < 0.05, ANOVA, Duncan's multiple range test). **B)** Proposed model for *B. amyloliquefaciens*–promoted cluster root formation of white lupin under LP conditions. The white lupin *LaYUC4* gene produces auxin. *B. amyloliquefaciens* SQR9 also produces auxin by its *ysnE* gene. Auxins produced by *LaYUC4* and SQR9 were pooled and transported together by root LaPIN2, which contributed to cluster root formation. Cluster roots can increase the uptake of nutrients and water under LP conditions in white lupin. WT, no inoculation; WT + SQR9, inoculated with SQR9; WT+*ΔysnE*, inoculated with *ΔysnE* mutant; TRV (WT), empty vector in noninoculated white lupin; TRV (WT) + SQR9, empty vector in white lupin inoculated with SQR9; TRV (WT) + *ΔysnE*, empty vector in white lupin inoculated with *ΔysnE*; TRV::*LaYUC4*, *LaYUC4*-silenced in noninoculated white lupin; TRV::*LaPIN2*, *LaPIN2*-silenced in noninoculated white lupin; TRV::*LaYUC4* + SQR9, *LaYUC4*-silenced in white lupin inoculated with SQR9; TRV::*LaPIN2* + SQR9, *LaPIN2*-silenced in white lupin inoculated with SQR9; NP, normal phosphorus; LP, low phosphorus; SQR9, *B. amyloliquefaciens* SQR9; *ΔysnE*, *ysnE*-deficit mutant in the SQR9 genetic background.

To further investigate the regulation pathway of auxin in SQR9-inoculated cluster root formation, we compared the results of the cluster root number between some treatments (Fig. 6A). First, the cluster root number of wild-type white lupin with SQR9 inoculation was significantly increased by 57.1% compared with noninoculated wild-type white lupin under low phosphorus conditions. However, cluster root number of wild-type white lupin with *ΔysnE* (the *ΔysnE* showed a 73.7% lower auxin content than SQR9) inoculation was significantly reduced by 27.3% compared with wild-type white lupin with SQR9 inoculation under low phosphorus conditions. These results showed that the SQR9-produced auxin was necessary for SQR9-promoting cluster root formation in white lupin. In addition, there was no significant difference in the number of cluster roots between empty vector in white lupin, TRV (WT), with SQR9-noninoculated and wild-type white lupin with SQR9-noninoculated under low phosphorus conditions, which showed that plasmid transformation operation did not affect the cluster root formation results. Also, the cluster root number of TRV (WT) with SQR9 inoculation was significantly increased by 57.8% compared with noninoculated TRV (WT) under low phosphorus conditions. Cluster root number of TRV (WT) with *ΔysnE* inoculation was significantly reduced by 30.7% compared with TRV (WT) with SQR9 inoculation under low phosphorus conditions. These results confirmed that the SQR9-produced auxin by its *ysnE* gene was essential for SQR9-promoting cluster root formation in white lupin.

Second, cluster root number of the *LaYUC4* knockdown plants (TRV::*LaYUC4*) was significantly reduced by 51.6% compared with TRV (WT) under low phosphorus conditions, which showed that white lupin *LaYUC4*-produced auxin could contribute to cluster root formation. However, under low phosphorus conditions, SQR9 inoculation significantly increased the cluster root number of TRV::*LaYUC4* by 43.5% compared with noninoculation. Next, cluster root number of the *LaPIN2* knockdown plants (TRV::*LaPIN2*) was significantly reduced by 82.1% compared with TRV (WT) under low phosphorus conditions, which showed that white lupin *LaPIN2*-transported auxin could greatly contribute to cluster root formation. Surprise, under low phosphorus, SQR9 inoculation could not increase the cluster root number of TRV::*LaPIN2*, which indicated that the white lupin auxin polar transport gene *LaPIN2* was essential for cluster root formation by integrating white lupin auxin synthesis gene *LaYUC4* and participating SQR9 under low phosphorus conditions.

Thus, our results showed that (i) white lupin *LaYUC4* gene produced auxin, which can contribute to cluster root formation; (ii) *B. amyloliquefaciens* SQR9 produced auxin by its *ysnE* gene, which is also important for cluster root formation; and (iii) the joint auxins produced by *LaYUC4* and SQR9 are pooled and transported together by the root *LaPIN2* gene, which launch cluster root formation in white lupin.

## Discussion

Efficient colonization by PGPR strains is a prerequisite for promoting plant growth (Qiu et al. 2012). *B. amyloliquefaciens* SQR9 is a rhizobacterium first isolated from the rhizosphere of cucumber (Cao et al. 2011). In our study, SQR9-GFP exhibited green fluorescence in roots (Supplementary Fig. S1), indicating that SQR9 could colonize white lupin roots. *Pseudomonas argentinensis* strain SA190 can induce root morphogenesis in *Arabidopsis* (Alwutayd et al. 2023). *Bacillus* improves the root system architecture of soybean (Esawi et al. 2018). In the present study, SQR9-inoculated white lupin showed significantly increased cluster root formation (Fig. 2B), consistent with *K. pneumoniae*–induced cluster root formation (Zhang et al. 2023). The addition of auxin can induce the development of lateral root primordium (Hirota et al. 2007; Ivanchenko et al. 2008). In addition, elevated endogenous auxin levels may lead to apoplast acidification, consequently promoting cell expansion in distal cells of the meristematic epidermis of roots (Barbez et al. 2017). Auxin levels increase in founder cell pairs of lateral roots before lateral root initiation (Dubrovsky et al. 2008). Zhang et al. (2023) reported that exogenous application of auxin can enhance cluster root formation in white lupin. In our study, auxin levels were significantly increased in the roots of SQR9-inoculated plants (Fig. 3D; Supplementary Fig. S3, A to C), confirming its essential role in this process. Auxin or auxin precursor L-tryptophan addition positively increased white lupin cluster root formation (Fig. 3B; Supplementary Fig. S2A). However, auxin synthesis inhibitor L-kynurenine or auxin transport inhibitor NPA addition inhibited white lupin cluster root formation (Fig. 3C; Supplementary Fig. S2B). These results suggest that auxin plays a key role in white lupin cluster root formation under phosphorus deficiency conditions. In addition, under NPA treatment (auxin transport inhibitor), even the application of SQR9 could not promote the increase of cluster root number under low phosphorus conditions (Fig. 3E), further confirming auxin is required for SQR9-promoting cluster root formation under low phosphorus conditions.

PGPRs, such as *Paenibacillus polymyxa, B. subtilis,* and *B. megaterium*, can regulate root development by modulating endogenous hormones in plants (Arkhipova et al. 2005; Verbon and Liberman 2016; Wang et al. 2021; Pantoja et al. 2023). Early studies on *A. thaliana* suggested that the key auxin biosynthetic gene *YUC4* was preferentially expressed in the pericycle founder cells and regulated lateral root formation (Stepanova et al. 2011; Tang et al. 2017). In our study, the relative expression of *LaYUC4* was significantly induced by SQR9 under low phosphorus (Fig. 5, A and B). Furthermore, cluster root formation of *LaYUC4* knockdown plants was significantly decreased relative to control plants carrying the empty vector under low phosphorus conditions with SQR9 inoculation (Fig. 6A), which also provides important evidence for cluster root formation via white lupin–produced auxin. In addition, auxin efflux regulators PIN-FORMED (PIN), mediates auxin distribution in lateral root initiation and development, are required for lateral root formation (Bennett et al. 1996; Benková et al. 2003). Previous studies suggested that application of PGPR could not promote lateral root formation under NPA treatment (Zamioudis et al. 2013; Li et al. 2021). In our study, the relative expression of *LaPIN2* in SQR9 inoculation roots was significantly upregulated. Moreover, cluster root formation of *LaPIN2* knockdown plants was significantly decreased relative to empty vector plants under low phosphorus conditions (Fig. 5H). And *LaPIN2* knockdown plants did not recover the cluster root formation even in SQR9 inoculation (Fig. 6A), indicating that the polar transport of auxin is essential for SQR9-promoting cluster root formation under low phosphorus conditions.

PGPR bacteria can directly stimulate plant growth through auxin biosynthesis (Asghar et al. 2002; Duca and Glick 2020). Bacteria can promote lateral root development by secreting auxin (Shah et al. 2022; Zhang et al. 2022a; Figueredo et al. 2023). In our study, the SQR9 showed strong auxin biosynthesis capability (Supplementary Fig. S5), suggesting that it may promote white lupin cluster root formation via auxin. The product of the *ysnE* gene is one of the most important intermediates in SQR9 auxin biosynthesis (Shao et al. 2015). In the present study, the *ΔysnE* showed significantly reduced cluster root formation (Fig. 4B), which confirmed the important role of SQR9-produced auxin in white lupin cluster root formation. The white lupin *LaYUC4* gene produces auxin. Also, *B. amyloliquefaciens* SQR9 produces auxin by its *ysnE* gene. After that, the joint auxins produced by *LaYUC4* and SQR9 are pooled and transported together by the root *LaPIN2* gene, which contributed to cluster root formation.

In conclusion, we show that the pooled auxins from white lupin *LaYUC4*-producing auxin and bacteria SQR9-producing auxin by its *ysnE* gene are transported by *LaPIN2* gene and then the LaPIN2-transported pooled auxins promoted cluster root formation under low phosphorus conditions (Fig. 6B). These findings enhance our understanding of how bacteria facilitate cluster root development with auxin mediation for higher phosphorus use in plants.

## Materials and methods

### Plant material and growth

White lupin (*L. albus* L.) accessions Amiga were used in the study as wild-type genotype. Seeds were surface sterilized with a 20% (v/v) ethanol solution for 10 min and then rinsed 3 times with distilled water. Following sterilization, the seeds were germinated in the moist kraft paper at room temperature for 3 d in the dark (Yu et al. 2021). The seedlings were grown in hydroponics boxes filled with sterile water, for 3 to 5 d (until a 4-cm radicle emerged). White lupin plants were grown in a nutrient solution (renewed every 4 d) as described by Le Thanh et al. (2021). Lupin plants were placed at a glasshouse with a 16-h light (26 °C)/8-h dark (22 °C), 40% (w/w) relative humidity, and a photosynthetic photon flux density of 150 mmol photons m^−2^ s^−1^. To explore the association between auxin and cluster root formation of white lupin under low phosphorus conditions, we utilized IAA (a type of auxin), L-tryptophan (a auxin precursor, L-try), L-kynurenine (a auxin biosynthesis inhibitor, L-kyn), and NPA (a auxin polar transport inhibitor). After 3 wk of transplanting, 0.5 and 1.0 *μ*m of IAA, 50  and 200 *μ*m of L-tryptophan, 10  and 30 *μ*m of L-kynurenine or 10, 20 , and 30 *μ*m of NPA were applied to plants for 5 d, and cluster root number was recorded at harvest (Zhang et al. 2023).

### White lupin root colonization

After grown in sterile water for 3 d, the lupin plants were soaked in GFP-tagged of *B. amyloliquefaciens* SQR9 suspension (1/4 sucrose-free MS medium, OD600 = 0.6) for 2 h at room temperature, and then the inoculated roots were washed with sterilized water (Liu et al. 2016). The plants were transplanted to half-strength nutrient solution for 7 d after inoculation (Zhang et al. 2023). The full strength of nutrient solution was used for 4 wk. The samples from roots (main roots, lateral roots, or cluster roots) were collected for observation under a confocal laser scanning microscope at the 488 nm excitation laser and 525/50 nm emission filter for GFP (intensity: 55%, gain: 700). *B. amyloliquefaciens* SQR9 or *ΔysnE* mutant inoculation was also used in the method.

### Measurement of total root length, cluster root number, and root fresh weight

The root samples were scanned using a flatbed scanner (Epson Perfection V700 Photo; Seiko Epson Corp., Nagano, Japan). The total root length was determined by WINRHIZO Reg 2016a, following the manufacturer's instructions (Regent Instruments Inc., Quebec, Canada). For determination the fresh weights, white lupin roots were thoroughly washed with deionized water 3 times and blotted dry with tissue paper. The number of cluster roots per plant was determined, including all developmental stages (young cluster root [YCR], mature cluster root [MCR], and old cluster root [OCR]). The number of cluster root per unit length was calculated by dividing the cluster root number by the total length (Zhang et al. 2023).

### Transcriptome analysis

The transcriptome basic data were obtained from White Lupin Genome Portal (www.whitelupin.fr). The processed reads were aligned to the genes of white lupin through the use of HISAT2 (version 2.2.1). To determine expression levels, the calculation was made based on the number of fragments per kilobase of transcript per million mapped reads. Differentially expressed genes (DEGs) were detected employing the DEseqR package, applying significance thresholds of |log2FC| ≥ 1, *P* < 0.05, and false discovery rate < 0.05. The annotation and categorization of DEGs were conducted with the aid of Mercator4 (version 2.0). Spearman's correlation analysis was executed in R (version 4.0.5) to analyze DEGs throughout the development of the cluster root according to transcriptomic data that spans 8 developmental stages of the cluster root (Hufnagel et al. 2020).

### RNA extraction and reverse transcription quantitative PCR analysis

Fresh root samples were crushed using a bead tissue lyser and homogenized to extract total RNA using TRIzol reagent following the manufacturer's instructions. In order to conduct the reverse transcription quantitative PCR (RT-qPCR) experiments, RNA from the samples was first reverse-transcribed into cDNA by utilizing the PrimeScript RT reagent kit with gDNA Eraser, in accordance with the manufacturer's guidelines. Following this, RT-qPCR analysis was carried out on a LightCycler Real-Time PCR Detection System (Bio-Rad) using an SYBR Green PCR Master Mix. The *UBQ10* gene served as the internal control for the expression analysis, and the primer sequences can be found in Supplementary Table S1. The data were collected from 3 replicates, and the relative expression levels were determined for each sample using the 2^−ΔΔ*Ct*^ method.

### *Agrobacterium rhizogenes*–mediated hairy root transformation

Seeds of white lupin were germinated and underwent surface sterilization as previously described. When the radicles reached a length of 10 mm, ∼3 mm segments from the tips were excised using a sterile scalpel. The remaining radicles were inoculated with the *A. rhizogenes* strain K599, which carries DR5rev::GFP vector (pGreenIIM DR5-n3GFP), and were cocultivated on MS (Caisson Labs, Smithfield, UT, USA) plates supplemented with 150 *μ*m acetosyringone for 3 d. Following this, the seedlings were transferred to a pot filled with half-strength nutrient solutions and cultivated for a duration of 7 d (Xia et al. 2024). White lupin carrying DR5rev::GFP was cultured in total nutrient solution for 3 wk with noninoculated or inoculated SQR9. The transgenic roots were performed through the detection of GFP fluorescent signals under a confocal laser scanning microscope at the 488 nm laser line.

### Construction of plant transformation vectors and VIGS

The pTRV1 and pTRV2 VIGS vectors were purchased from YouBio (Biotechnology Company, Changsha, China). The 240-bp fragment of *LaYUC4* (Lalb_Chr14g0361961) and 259-bp fragment of *LaPIN2* (Lalb_Chr12g0199691) were amplified from root cDNA of white lupine and inserted into the pTRV2 vector digested by XbaI to generate pTRV2-*LaYUC4* and pTRV2-*LaPIN2*. Primers were listed in Supplementary Table S1. Tobacco rattle virus (TRV)–infected plants were used as viral vector controls. *A. tumefaciens* GV3101 carrying pTRV1 or pTRV2 derivatives were cultured in LB medium with Kana antibiotics at 28 °C until OD600 of 0.8. The cells were collected and resuspended in buffer solution (10 mm 2-[*N*-morpholino] ethanesulfonic acid, 0.1 mm acetosyringone, and 10 mm MgCl_2_) to reach an OD600 of 0.5 and incubated at room temperature for 2 h. For infection, *A. tumefaciens* GV3101 carrying derivatives of pTRV1 and pTRV2 were mixed at a 1:1 ratio and inoculated. Then, the plant roots were placed in the mixed bacterial solution for 2 h at 26 ℃ (Aslam et al. 2022).

### IAA and sucrose concentration analysis

IAA was extracted from the roots and *B. amyloliquefaciens* SQR9, and sucrose was extracted from the roots. Measurement of IAA and sucrose was conducted by HPLC according to the studies by Xu et al. (2022) and Xia et al. (2024). Briefly, the roots were frozen in liquid nitrogen after determining fresh weight. Then, the IAA and sucrose measurement were carried out using HPLC. The standard IAA sample and standard sucrose sample were bought from Sigma-Aldrich (St Louis, MO, USA). For the measurement of IAA in *B. amyloliquefaciens* SQR9, the supernatant was obtained by passing through filter paper and then measured IAA by HPLC according to the study by Xu et al. (2023).

### Accession numbers

Sequence data for the *LaYUC4* and *LaPIN2* from this article can be found in the White Lupin Genome data libraries (https://www.whitelupin.fr/index.html) under accession numbers: Lalb_Chr14g0361961 and Lalb_Chr12g0199691.

## Supplementary Material

kiae676_Supplementary_Data

## Data Availability

Data supporting the findings of this work are available within the paper and its supplemental figures and tables. The raw reads of the transcriptome analysis were downloaded from Hufnagel et al. (2020) (White Lupin Genome Portal, Peret Lab, https://www.whitelupin.fr/download.html). The datasets generated and analysed in the current study are available from the corresponding author on reasonable request.

## Supplementary data

The following materials are available in the online version of this article.

**Supplementary Figure S1.** *B. amyloliquefaciens* SQR9 can colonize white lupin roots.

**Supplementary Figure S2.** Exogenous application of L-tryptophan or L-kynurenine to white lupin under normal phosphorus and low phosphorus conditions.

**Supplementary Figure S3.** The auxin concentration in white lupin roots increased under low phosphorus with SQR9 inoculation.

**Supplementary Figure S4.** Sucrose content in white lupin roots with or without SQR9 inoculation under normal phosphorus and low phosphorus conditions.

**Supplementary Figure S5.** IAA content of *B. amyloliquefaciens* SQR9 and the mutant *ΔysnE*.

**Supplementary Figure S6.** Heatmap of DEGs in roots subjected to different treatments under low phosphorus.

**Supplementary Table S1.** List of primers.

## Dive Curated Terms

The following phenotypic, genotypic, and functional terms are of significance to the work described in this paper:

UBQ10 Gramene: AT4G05320
UBQ10 Araport: AT4G05320

VIGS Gramene: Virus-induced gene silencing
VIGS Araport: Virus-induced gene silencing

auxin CHEBI: CHEBI:22676

cluster CHEBI: CHEBI:33731

YUC4 Gramene: AT5G11320
YUC4 Araport: AT5G11320
