# Supplementary figures and images for: Bacillus amyloliquefaciens promotes cluster root formation of white lupin under low phosphorus by mediating auxin levels
Source: Plant Physiol. 2024 Dec 25;197(2):kiae676. doi: 10.1093/plphys/kiae676 (PMC11831804; doi:10.1093/plphys/kiae676)

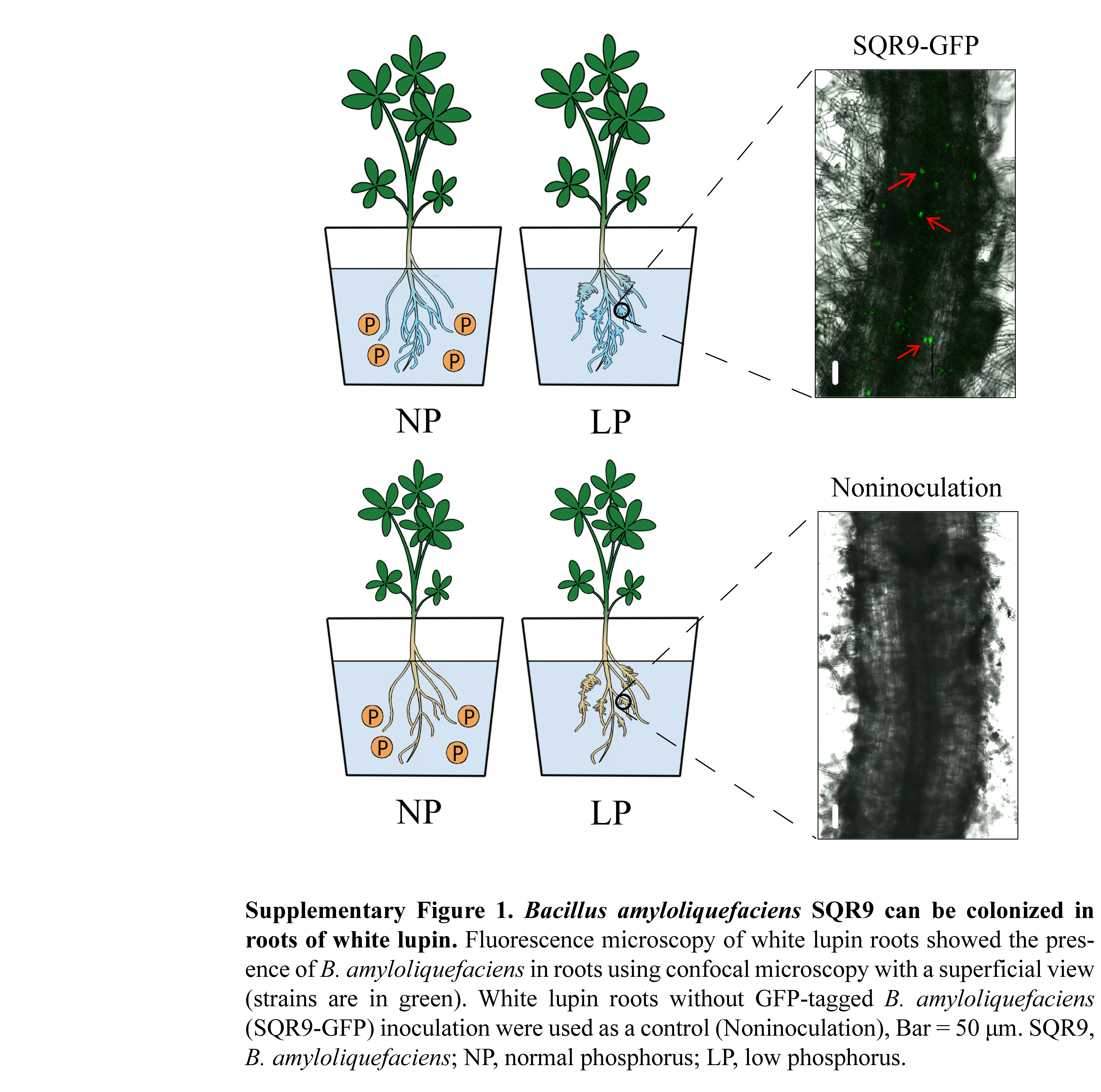

Supplement: kiae676_Supplementary_Data [file kiae676_supplementary_data.zip › Fig S1 .tif]

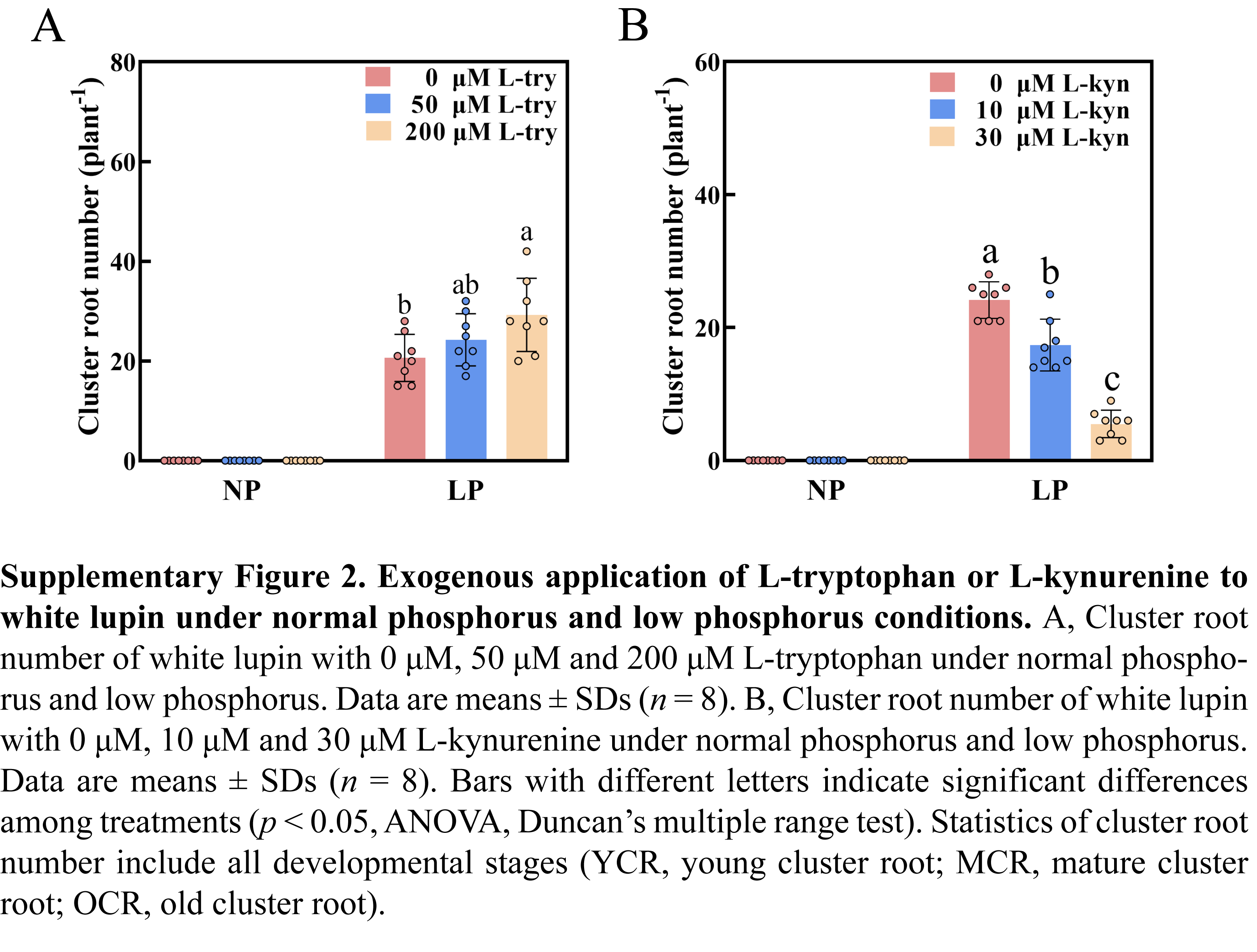

Supplement: kiae676_Supplementary_Data [file kiae676_supplementary_data.zip › Fig S2 .tif]

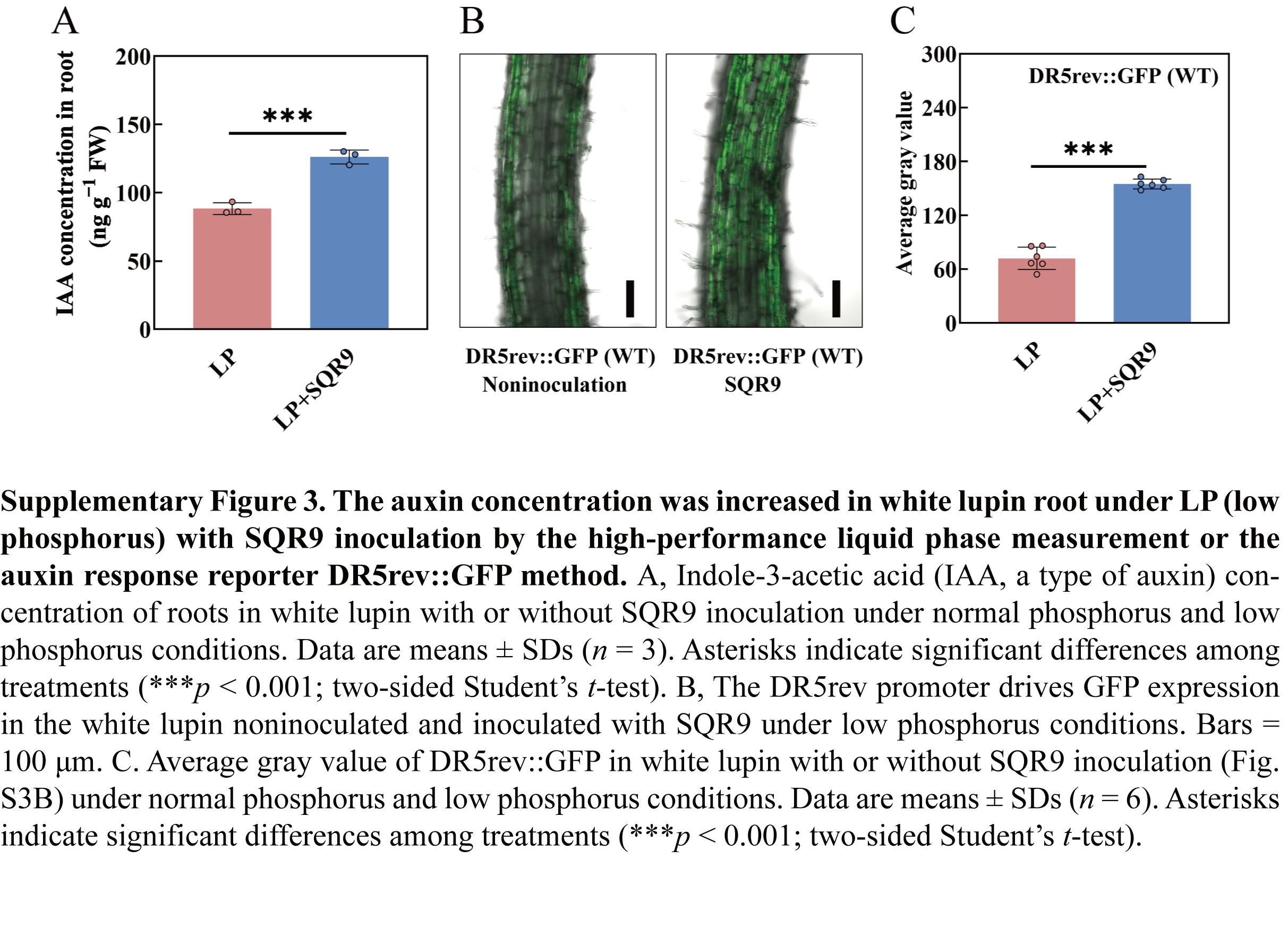

Supplement: kiae676_Supplementary_Data [file kiae676_supplementary_data.zip › Fig S3 .tif]

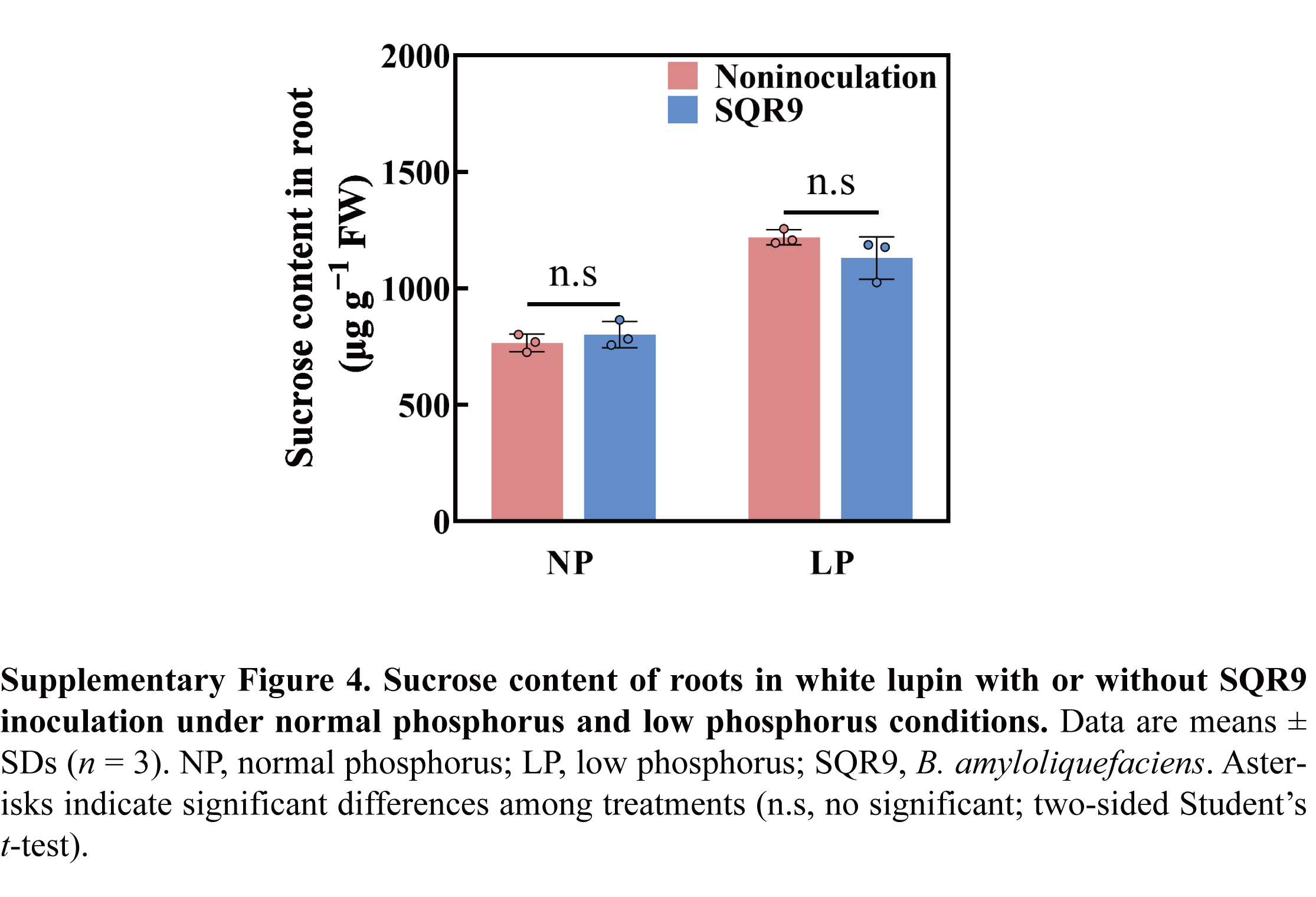

Supplement: kiae676_Supplementary_Data [file kiae676_supplementary_data.zip › Fig S4 .tif]

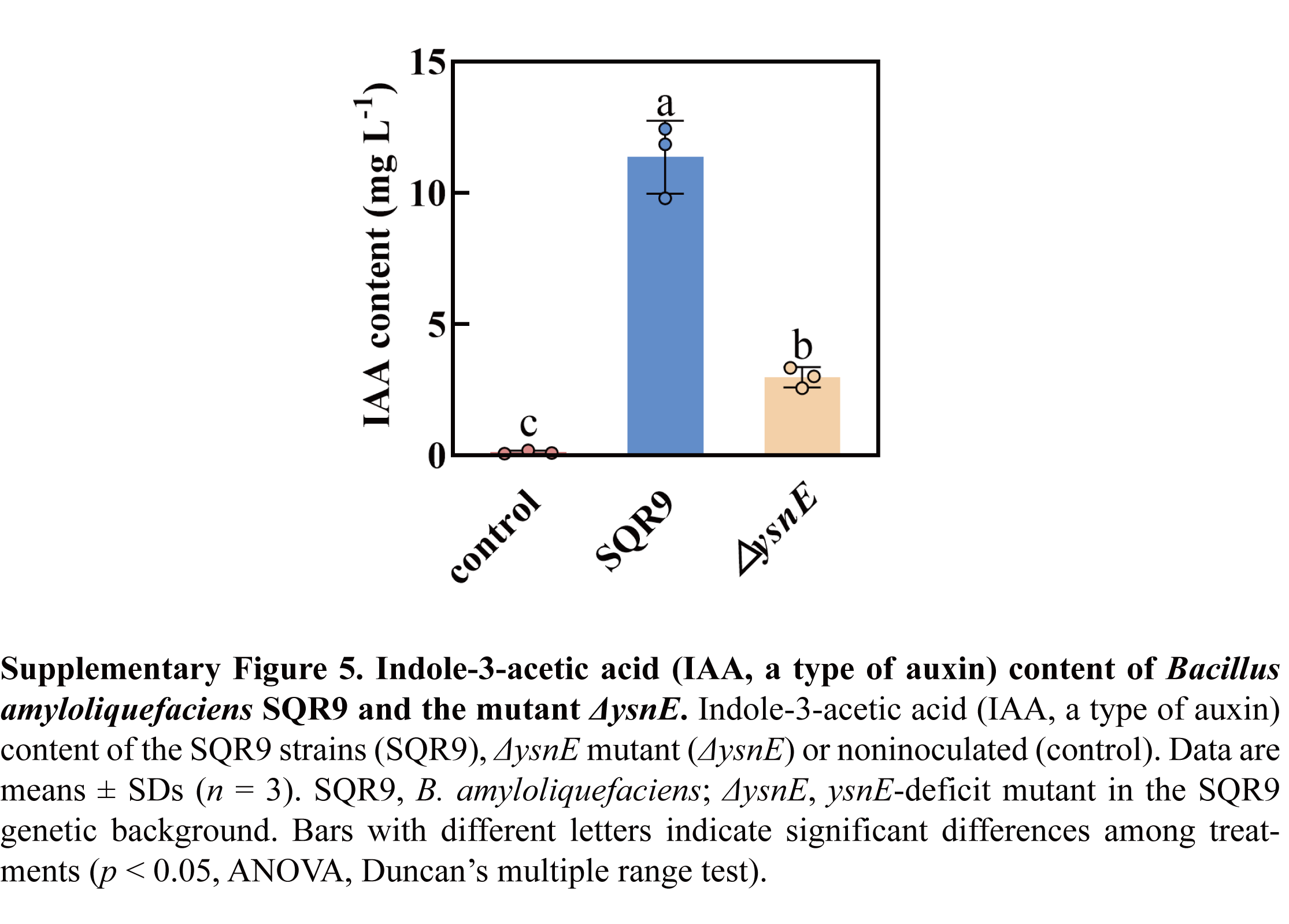

Supplement: kiae676_Supplementary_Data [file kiae676_supplementary_data.zip › Fig S5 .tif]

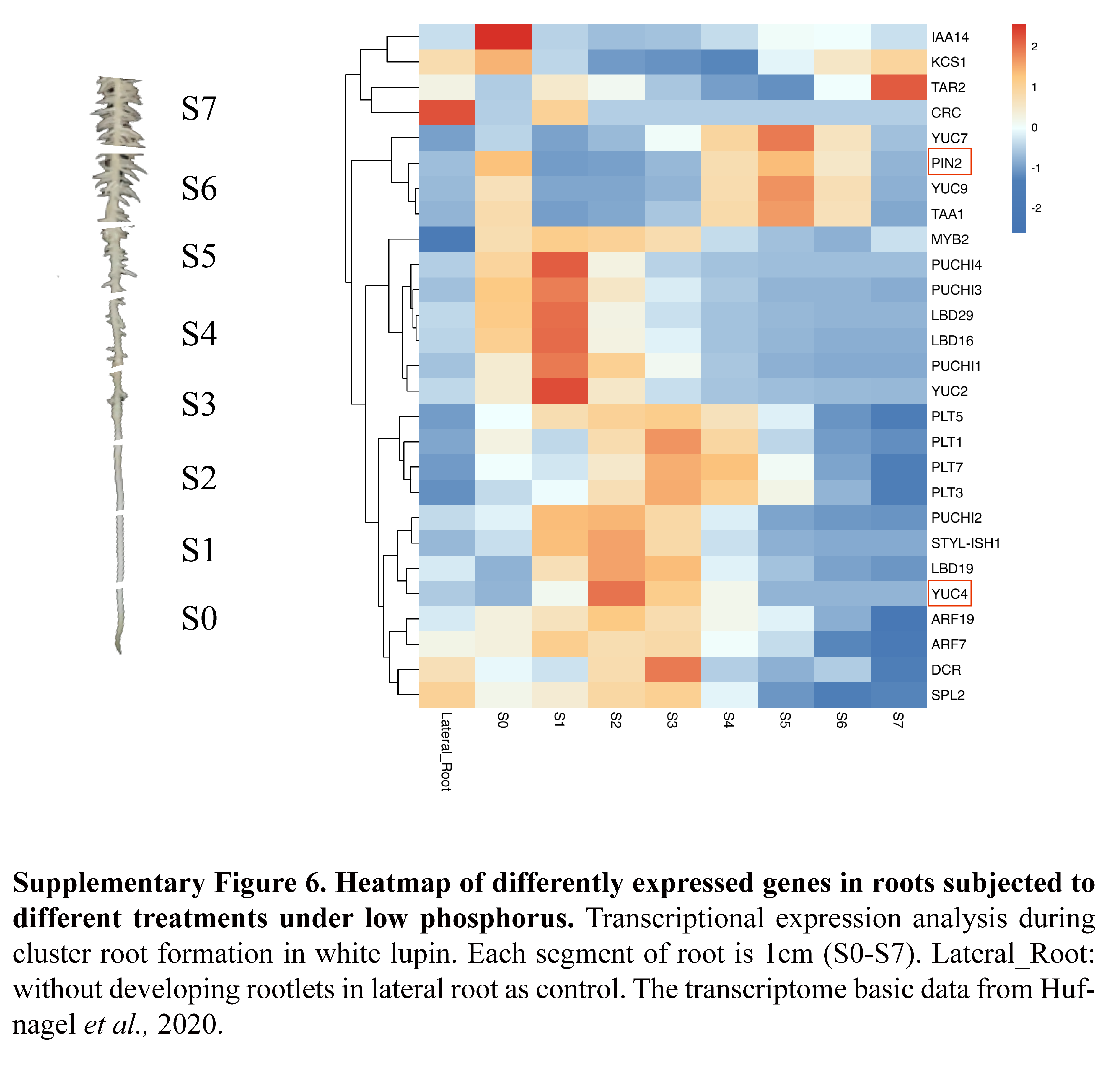

Supplement: kiae676_Supplementary_Data [file kiae676_supplementary_data.zip › Fig S6 .tif]
