# Supplementary material for: Bacillus amyloliquefaciens promotes cluster root formation of white lupin under low phosphorus by mediating auxin levels
Source: Plant Physiol. 2024 Dec 25;197(2):kiae676. doi: 10.1093/plphys/kiae676 (PMC11831804; doi:10.1093/plphys/kiae676)
Supplement: kiae676_Supplementary_Data [file pp.02780.2024-s11.pdf › pp.02780.2024-s11.pdf]

Plant Physiology®

B.amyloliquefaciens promotes the cluster root formation of white lupin under low phosphorus through auxins mediation

Jinyong Yang, Shenglan Li, Xiangxue Zhou, Chongxuan Du, Ju Fang, Xing Li, Jun Zhao, Fan Ding, Yue Wang, Qian Zhang, Zhengrui Wang, Jianping Liu, Gangqiang Dong, Jianhua Zhang, Feiyun Xu, and Weifeng Xu

Review Timeline:

Submission Date:	26-Apr-2024
Editorial Decision:	14-May-2024
Resubmission Received:	07-Nov-2024
Accepted:	26-Nov-2024

14-May-2024

Prof. Dr. Weifeng Xu
福建农林大学
Jinshan Fuzhou, orcid|||||||
China

RE: Bacillus amyloliquefaciens promotes the cluster root formation through auxin mediation from white lupin or bacterium

Dear Dr. Weifeng Xu:

Thank you for giving us the opportunity to review your manuscript for publication in the Plant Physiology. The reviewers have found the manuscript of interest, but have raised a number of concerns that will require your attention. As these concerns are of some gravity and will entail substantial changes to the manuscript, you are likely to need additional time to address them. I must therefore reject the manuscript in its present form. Nonetheless, if you are able to address the points raised in review, I would welcome your resubmission of a manuscript that incorporates your responses.

In particular, I direct your attention to the following comments for consideration in a resubmitted manuscript.

1) Increased endogenous auxin contents in SQR9-inoculated plants are simply caused by SQR9-induced transcription of YUC genes in white lupin or influx of rhizospheric bacteria SQR9-synthesized auxin. Authors should clarify and revise the confusing title of the manuscript.
2) What's are the possible components acts upstream and downstream of auxin in regulating SQR9-mediated cluster root formation?
3) SQR9 mainly increases transcriptional expression of auxin biosynthesis gene YUCs. In stead of auxin transport inhibitor NPA, authors should detect the effects of auxin biosynthesis inhibitor L-kynurenine and auxin precursor L-tryptophan on SQR9-meidated cluster root formation under low phosphorus.
4) As shown in Figure 2, SQR9 specifically induces cluster root formation under low phosphorus but not under normal phosphorus conditions. The situations in response to normal phosphorus are important controls for all other studies in Figures 3, 4 and 5. Authors should include the relevant data in the manuscript.
5) In addition to the direct auxin measurements, it's better also to visualize auxin distribution in roots of white lupin using the auxin response reporters, like DR5rev:GFP.
6) Figure 5E lacks controls. Author should also quantify cluster root numbers of SQR9- and ΔysnE mutant-inoculated plants with empty vector.
7) Only 4 plants are used to quantify cluster root numbers in the manuscript. To make data more solid, authors should increase the amount of plants for quantifications.

Please note that any resubmission will be treated as a new entry to the system and is likely to go out for review.

If you do have any queries or concerns, you are welcome to contact me in the first instance.

Thank you again for allowing Plant Physiology to review your work. We look forward to hearing from you soon.

Sincerely,

Editor, Plant Physiology

-- Reviewer comments:
Reviewer #1 (Comments for the Author):

The MS by Yang et al. describes the results: 1) The plant-beneficial Bacillus amyloliquefaciens SQR9 promotes cluster root formation under low phosphorus conditions. 2) SQR9 inoculation increase endogenous auxin levels in white lupin. 3) Auxin is required in the process of SQR9-regulated cluster root formation. However, the underlying regulatory mechanism is still not understood. It has been reported that auxin is indispensable for B. amyloliquefaciens-induced lateral root development in Arabidopsis (Li et al., 2021). Therefore, demonstrating SQR9 promotes cluster root formation by increasing auxin contents under low phosphorus conditions in white lupin is not so surprising. In addition, I would be grateful if the authors could clarify the following points:
Major comments:
1) Increased endogenous auxin contents in SQR9-inoculated plants are simply caused by SQR9-induced transcription of YUC genes in white lupin or influx of rhizospheric bacteria SQR9-synthesized auxin. Authors should clarify and revise the confusing

title of the manuscript.
2) What's are the possible components acts upstream and downstream of auxin in regulating SQR9-mediated cluster root formation?
3) SQR9 mainly increases transcriptional expression of auxin biosynthesis gene YUCs. In stead of auxin transport inhibitor NPA, authors should detect the effects of auxin biosynthesis inhibitor L-kynurenine and auxin precursor L-tryptophan on SQR9-meidated cluster root formation under low phosphorus.
4) As shown in Figure 2, SQR9 specifically induces cluster root formation under low phosphorus but not under normal phosphorus conditions. The situations in response to normal phosphorus are important controls for all other studies in Figures 3, 4 and 5. Authors should include the relevant data in the manuscript.
5) In addition to the direct auxin measurements, it's better also to visualize auxin distribution in roots of white lupin using the auxin response reporters, like DR5rev:GFP.
6) Figure 5E lacks controls. Author should also quantify cluster root numbers of SQR9- and ΔysnE mutant-inoculated plants with empty vector.
7) Only 4 plants are used to quantify cluster root numbers in the manuscript. To make data more solid, authors should increase the amount of plants for quantifications.

Minor comments:
1) The manuscript requires some improvement in English language usage.
2) In additional to quantified data, please at least show one representative image of the cluster roots phenotype.

Reviewer #2 (Comments for the Author):

Comments to the Author:
The manuscript entitled "Bacillus amyloliquefaciens promotes the cluster root formation under low phosphorus through auxin mediation from white lupin or bacterium" by Yang et al revealed that SQR9 promotes the cluster root formation under low phosphorus. The manuscript consists of a total of 5 main figures, including 4 figures describing phenotypic results and 1 schematic diagram of the proposed approach, without any mechanistic analysis. Furthermore, based on this study, SQR9 promotes the cluster root formation is largely result from its synthesized auxin, which is not surprising since auxin is a key plant hormone in root development. Furthermore, the logic and writing of this manuscript are poor, which need to be improved. Here are the main comments:
1. Fig. 2B, 3B-D, 4B, and 5D, E should show phenotypic figures, not just statistical results.
2. The relative expression of YUC2, YUC3, YUC7, YUC9, and PIN2 in SQR9-inoculation roots was significantly up-regulated according to Fig. 5A, B, why only LaYUC4 knockdown plants TRV::LaYUC4 was significantly decreased the cluster root number under low phosphorus compared with empty vector control according to Fig. 5D?
3. YUC4 is an auxin synthesis gene, which increases auxin content. External auxin application promotes the formation of cluster root under low phosphorus conditions according to Fig. 3C. Additionally, the increased auxin content in SQR9 also contributes to the promotion of cluster root formation under low phosphorus conditions, as depicted in Figure 4. However, the involvement of the LaYUC4 gene from white lupin in the SQR9-mediated promotion of cluster root formation under low phosphorus conditions, as indicated in Figure 5E. how to explain this result?
4. NPA inhibited the number of cluster root under low phosphorus conditions according to Fig. 3B, indicating that the polar transport of auxin in white lupin plays a crucial role in the formation of cluster root. And under NPA treatment, even the application of SQR9 could not promote the increase of cluster root number under low phosphorus conditions according to Fig. 3D. Therefore, SQR9 produced auxin might be absorbed by plants through polar auxin transport. Authors need to investigate which auxin transport might be involved.
5. There is no normal phosphorus treatment control in Figures 3A, 4A, and 4C. And the difference in fresh root weight was not related to low phosphorus conditions according to Fig. 2D.
6. Given the increase in cluster root numbers under low phosphorus conditions, as depicted in Figure 2B, it remains inconclusive whether the rise in IAA content in roots, as shown in Figure 3A, is directly attributable to SQR9.
7. In line 573-575, SQR9 may not only be expressed in cortical cells according to Fig. 1. Is SQR9 only colonized in the cluster root? How do you explain that? The fluorescence signal of SQR9-GFP could also be detected in other roots under normal and low phosphorus conditions.
8. According to Figure 2A, SQR9 promoted total root length under normal phosphorus conditions, but inhibited total root length under low phosphorus compared with normal phosphorus conditions. The author should explain this result.
9. In line 154-156, the relative expression of IAA27 and PIN3 in SQR9-inoculation roots were not increased according to Fig. 5A, the description is incorrect.
10. The author should explain the meaning of cluster root in the introduction.
11. In line 81-83, sucrose may contribute to the cluster root formation in white lupin under low phosphorus conditions, the authors could examine whether Bacillus amyloliquefaciens SQR9 affected sugar content.

Other comments:
1) Why are the statistical differences between Fig. 5A and 5B inconsistent?
2) In line 201-204, the conclusion is not accurate.

3) In line 163, the author forgot to label Fig. 5B.
4) Why put "or bacterium" at the end of the title?
5) In many instances, the results descriptions only describe the experimental results without presenting the corresponding conclusions.

To: Editor of *Plant Physiology*,

30th October 2024

Dear Prof. Dr.,

Your Ref: Title: "*Bacillus amyloliquefaciens* promotes the cluster root formation through auxin mediation from white lupin or bacterium" (PP2024-RA-00622).

Thank you very much for your email dated 14th May 2024 regarding our manuscript above. We are very pleased that our paper could be accepted in *Plant Physiology* after the careful revision. We are very grateful to you and the referees for the constructive comments. We have revised our manuscript point by point to address the comments from editor and referee with new experimental data. Revised portions are marked in blue on the paper. Our responses and modifications are listed below:

Comments from the Editor:

Question#1: Increased endogenous auxin contents in SQR9-inoculated plants are simply caused by SQR9-induced transcription of YUC genes in white lupin or influx of rhizospheric bacteria SQR9-synthesized auxin. Authors should clarify and revise the confusing title of the manuscript.

Answer: Thank you very much for your good comments. The *ysnE* is a key gene for auxin biosynthesis in SQR9. We found that *ysnE*-defective of SQR9 mutant inoculation could not significantly increase cluster root number in white lupin under low phosphorus conditions (Figs. 4A, B and Fig. 6A). Furthermore, cluster root number of the *LaYUC4* (a auxin biosynthesis gene in white lupin) knockdown plants (TRV::*LaYUC4*) with SQR9 inoculation was significantly reduced compared with wild-type white lupin with SQR9 inoculation under low phosphorus conditions (Fig. 6A). In addition, cluster root number of the *LaPIN2* (a auxin transport gene in white lupin) knockdown plants (TRV::*PIN2*) with SQR9 inoculation was significantly

reduced compared with wild-type white lupin with SQR9 inoculation under low phosphorus conditions (Fig. 6A).

Firstly, cluster root number of wild-type white lupin with SQR9 inoculation was significantly higher than that of noninoculated wild-type white lupin under low phosphorus conditions. However, cluster root number of wild-type white lupin with *ΔysnE* (The *ΔysnE* showed a 73.7% lower auxin content than SQR9) inoculation was significantly lower than that of wild-type white lupin with SQR9 inoculation under low phosphorus conditions (Fig. 6A and Fig. S5). These results showed that the SQR9-produced auxin was necessary for SQR9-promoting cluster root formation in white lupin. Secondly, cluster root number of the *LaYUC4* knockdown plants (TRV::*LaYUC4*) with SQR9 inoculation was significantly reduced compared with wild-type white lupin or TRV (WT) with SQR9 inoculation under low phosphorus conditions (Fig. 6A), which showed that the white lupin *LaYUC4*-produced auxin was also necessary for cluster root formation with participanting-SQR9. Thirdly, cluster root number of the *LaPIN2* knockdown plants (TRV::*PIN2*) with SQR9 inoculation was significantly reduced compared with wild-type white lupin with SQR9 inoculation under low phosphorus conditions (Fig. 6A). These results showed that the white lupin auxin polar transport gene *LaPIN2* is essential for cluster root formation with participanting-SQR9 under low phosphorus conditions. Finally, cluster root number of the TRV::*PIN2* with SQR9 inoculation was significantly lower than that of TRV::*LaYUC4* with SQR9 inoculation or wild-type white lupin with SQR9 (*ysnE* gene contributes to SQR9 auxin production) inoculation under low phosphorus conditions (Fig. 6A). Taken together, we proposed that pooled-auxins from white lupin *LaYUC4*-producing auxin and bacteria SQR9-producing auxin by its *ysnE* gene were transported by *LaPIN2* gene and then the LaPIN2-transported pooled-auxins promote cluster root formation under low phosphorus conditions (Fig. 6B). So, the title has been revised to "*Bacillus amyloliquefaciens* promotes the cluster root formation of white lupin under low phosphorus through auxins mediation" (Lines 1-2, Lines 44-51, Line 167, Lines 169-171, Lines 205-242, Lines 304-311, Lines 519-529, Lines 550-569, Lines 812-822, Lines 844-863, Lines 906-911).

A

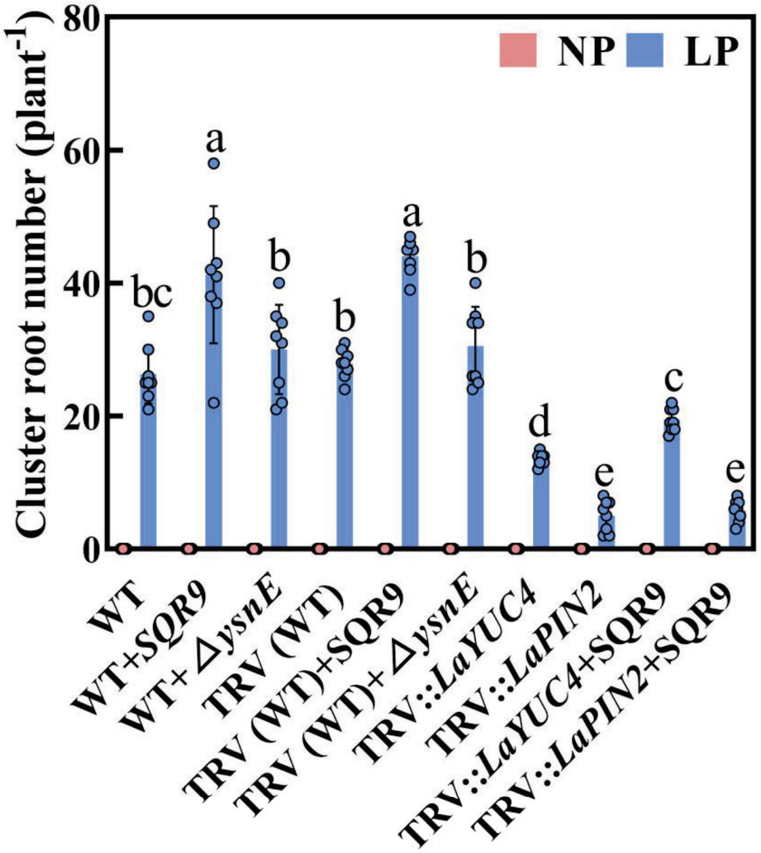

B

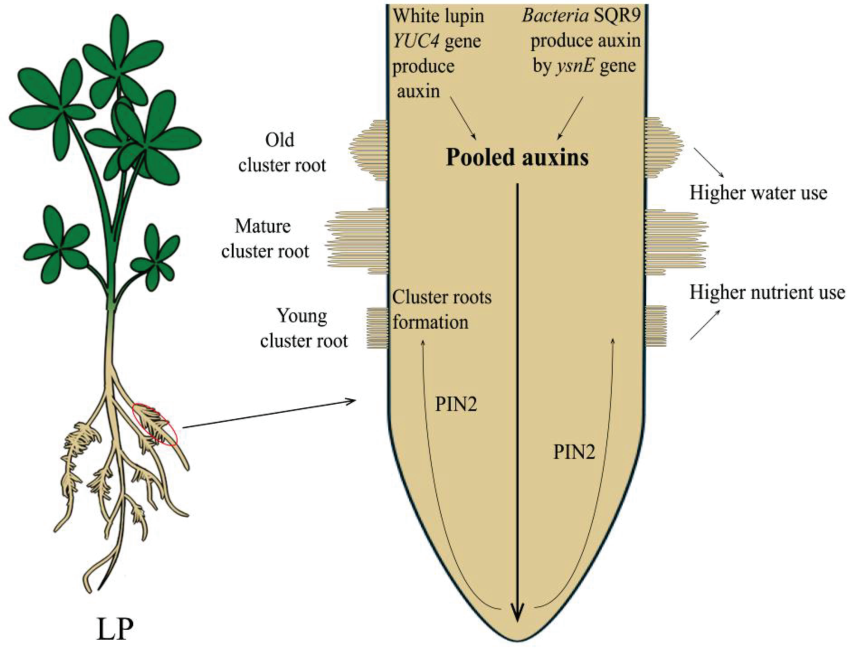

Figure 6. ***Bacillus amyloliquefaciens*** **promotes the cluster root formation of white lupin under low phosphorus through auxins mediation (auxin biosynthesis and**

transport).

Question#2: What's are the possible components acts upstream and downstream of auxin in regulating SQR9-mediated cluster root formation?

Answer: The *ysnE* is a key gene for auxin biosynthesis in SQR9. We found that *ysnE*-defective of SQR9 mutant inoculation could not significantly increase cluster root number in white lupin under low phosphorus conditions (Figs. 4A, B and Fig. 6A). Furthermore, cluster root number of the *LaYUC4* (a auxin biosynthesis gene in white lupin) knockdown plants (TRV::*LaYUC4*) with SQR9 inoculation was significantly reduced compared with wild-type white lupin with SQR9 inoculation under low phosphorus conditions (Fig. 6A). In addition, cluster root number of the *LaPIN2* (a auxin transport gene in white lupin) knockdown plants (TRV::*PIN2*) with SQR9 inoculation was significantly reduced compared with wild-type white lupin with SQR9 inoculation under low phosphorus conditions (Fig. 6A). So, the auxin synthesis gene *LaYUC4* in white lupin and auxin-synthesis gene *ysnE* in SQR9 could be the upstream component in regulating SQR9-mediated cluster root formation. Auxin transport gene *LaPIN2* could be the downstream component in regulating SQR9-mediated cluster root formation. As shown in Figure 6B, the pooled-auxins from white lupin *LaYUC4*-producing auxin and bacteria SQR9-producing auxin by its *ysnE* gene were transported by *LaPIN2* gene and then the LaPIN2-transported pooled-auxins promote cluster root formation under low phosphorus conditions (Lines 167, Lines 207-242, Lines 304-311, Lines 519-529, Lines 550-569, Lines 812-822, Lines 844-863).

Question#3: SQR9 mainly increases transcriptional expression of auxin biosynthesis gene YUCs. In stead of auxin transport inhibitor NPA, authors should detect the effects of auxin biosynthesis inhibitor L-kynurenine and auxin precursor L-tryptophan on SQR9-meidated cluster root formation under low phosphorus.

Answer: Thank you for the valuable comments. We added auxin biosynthesis inhibitor L-kynurenine and auxin precursor L-tryptophan experiments to validate the interaction between auxin and cluster root formation. The results showed that L-tryptophan addition positively increased white lupin cluster root formation. However, the L-kynurenine addition inhibited white lupin cluster root formation (Lines 144-155, Lines 262-265, Lines 877-884 and Fig. S2).

Question#4: As shown in Figure 2, SQR9 specifically induces cluster root formation under low phosphorus but not under normal phosphorus conditions. The situations in response to normal phosphorus are important controls for all other studies in Figures 3, 4 and 5. Authors should include the relevant data in the manuscript.

Answer: Many thanks. Yes, we did this as suggested. We re-conducted the relevant experiments under normal phosphorus supply and low phosphorus conditions (Figs. 3A, 4A and Fig. 6A). And, the relevant data have been added in the revised manuscript (Figs. 3B-C, 4B-C and Fig. 6A).

Question#5: In addition to the direct auxin measurements, it's better also to visualize auxin distribution in roots of white lupin using the auxin response reporters, like DR5rev:GFP.

Answer: Thanks for your comments. Yes, we did this as suggested. We used the auxin response reporter DR5rev::GFP plasmid and transferred it into white lupin. The results of auxin measured by high-performance liquid phase showed that the indole-3-acetic acid (IAA, a type of auxin) concentration of roots in white lupin with SQR9 inoculation was significantly increased compared to the uninoculated plants under low phosphorus conditions (Fig. S3A). DR5rev::GFP fluorescence (Figs. S3B, C) also showed that the indole-3-acetic acid concentration of roots in white lupin with SQR9 inoculation was significantly increased. Both the high-performance liquid phase auxin measurement and the auxin response reporter DR5rev::GFP method can

accurately determine the auxin concentration in white lupin root under LP (low phosphorus) with SQR9 inoculation (Lines 157-159, Lines 260-261, Lines 889-897 and Figs. S3A-C).

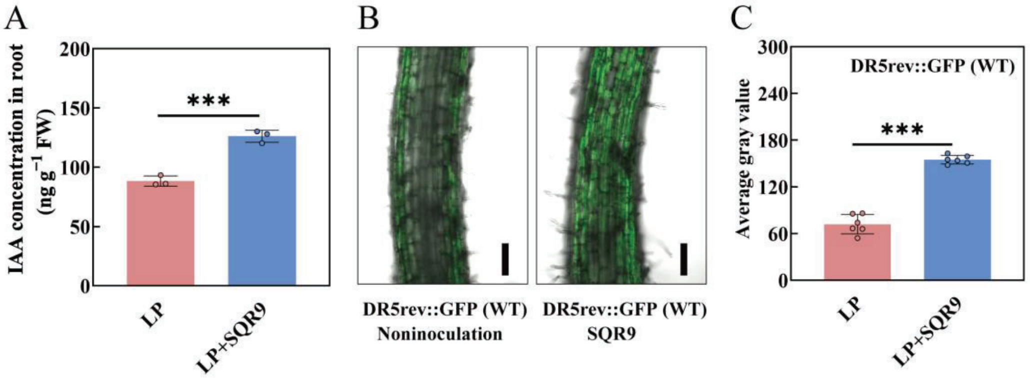

Supplementary Figure 3. The auxin concentration was increased in white lupin root under LP (low phosphorus) with SQR9 inoculation by the high-performance liquid phase measurement or the auxin response reporter DR5rev::GFP method.

Question#6: Figure 5E lacks controls. Author should also quantify cluster root numbers of SQR9- and *ΔysnE* mutant-inoculated plants with empty vector.

Answer: Many thanks for your comments! Yes, we added the controls in the adjusted Fig. 6A. TRV (WT) means the wild-type white lupin with empty vector. The cluster root numbers of TRV (WT) under LP or SQR9 or *ΔysnE* mutant was almostly same to that of wild-type white lupin without empty vector.

Question#7: Only 4 plants are used to quantify cluster root numbers in the manuscript. To make data more solid, authors should increase the amount of plants for quantifications.

Answer: Many thanks for your constructive comments! To make data more solid, we had increased to 6-8 plants for quantifications in the revised manuscript (Figs 2, 3, 4, 5, 6 and Fig. S2).

Comments from the Referee#1:

Question#1: Increased endogenous auxin contents in SQR9-inoculated plants are simply caused by SQR9-induced transcription of YUC genes in white lupin or influx of rhizospheric bacteria SQR9-synthesized auxin. Authors should clarify and revise the confusing title of the manuscript.

Answer: Thank you very much for your good comments. The *ysnE* is a key gene for auxin biosynthesis in SQR9. We found that *ysnE*-defective of SQR9 mutant inoculation could not significantly increase cluster root number in white lupin under low phosphorus conditions (Figs. 4A, B and Fig. 6A). Furthermore, cluster root number of the *LaYUC4* (a auxin biosynthesis gene in white lupin) knockdown plants (TRV::*LaYUC4*) with SQR9 inoculation was significantly reduced compared with wild-type white lupin with SQR9 inoculation under low phosphorus conditions (Fig. 6A). In addition, cluster root number of the *LaPIN2* (a auxin transport gene in white lupin) knockdown plants (TRV::*PIN2*) with SQR9 inoculation was significantly reduced compared with wild-type white lupin with SQR9 inoculation under low phosphorus conditions (Fig. 6A).

Firstly, cluster root number of wild-type white lupin with SQR9 inoculation was significantly higher than that of noninoculated wild-type white lupin under low phosphorus conditions. However, cluster root number of wild-type white lupin with *ΔysnE* (The *ΔysnE* showed a 73.7% lower auxin content than SQR9) inoculation was significantly lower than that of wild-type white lupin with SQR9 inoculation under low phosphorus conditions (Fig. 6A and Fig. S5). These results showed that the SQR9-produced auxin was necessary for SQR9-promoting cluster root formation in white lupin. Secondly, cluster root number of the *LaYUC4* knockdown plants (TRV::*LaYUC4*) with SQR9 inoculation was significantly reduced compared with wild-type white lupin or TRV (WT) with SQR9 inoculation under low phosphorus conditions (Fig. 6A), which showed that the white lupin *LaYUC4*-produced auxin was also necessary for cluster root formation with participanting-SQR9. Thirdly, cluster

root number of the *LaPIN2* knockdown plants (TRV::*PIN2*) with SQR9 inoculation was significantly reduced compared with wild-type white lupin with SQR9 inoculation under low phosphorus conditions (Fig. 6A). These results showed that the white lupin auxin polar transport gene *LaPIN2* is essential for cluster root formation with participanting-SQR9 under low phosphorus conditions. Finally, cluster root number of the TRV::*PIN2* with SQR9 inoculation was significantly lower than that of TRV::*LaYUC4* with SQR9 inoculation or wild-type white lupin with SQR9 (*ysnE* gene contributes to SQR9 auxin production) inoculation under low phosphorus conditions (Fig. 6A). Taken together, we proposed that pooled-auxins from white lupin *LaYUC4*-producing auxin and bacteria SQR9-producing auxin by its *ysnE* gene were transported by *LaPIN2* gene and then the LaPIN2-transported pooled-auxins promote cluster root formation under low phosphorus conditions (Fig. 6B). So, the title has been revised to "*Bacillus amyloliquefaciens* promotes the cluster root formation of white lupin under low phosphorus through auxins mediation" (Lines 1-2, Lines 44-51, Line 167, Lines 169-171, Lines 205-242, Lines 304-311, Lines 519-529, Lines 550-569, Lines 812-822, Lines 844-863, Lines 906-911).

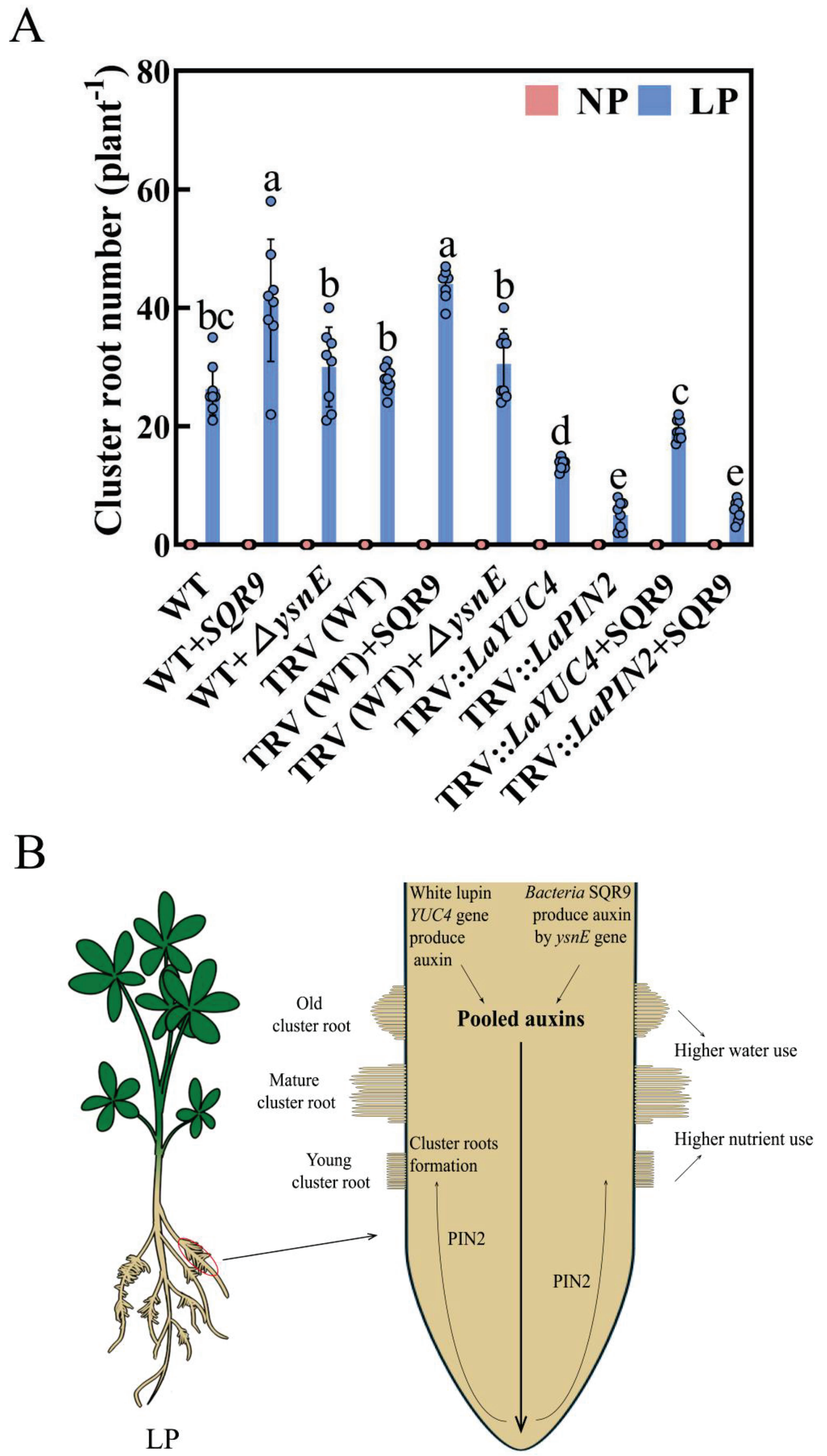

Figure 6. ***Bacillus amyloliquefaciens*** **promotes the cluster root formation of white lupin under low phosphorus through auxins mediation. (auxin biosynthesis and**

transport).

Question#2: What's are the possible components acts upstream and downstream of auxin in regulating SQR9-mediated cluster root formation?

Answer: The *ysnE* is a key gene for auxin biosynthesis in SQR9. We found that *ysnE*-defective of SQR9 mutant inoculation could not significantly increase cluster root number in white lupin under low phosphorus conditions (Figs. 4A, B and Fig. 6A). Furthermore, cluster root number of the *LaYUC4* (a auxin biosynthesis gene in white lupin) knockdown plants (TRV::*LaYUC4*) with SQR9 inoculation was significantly reduced compared with wild-type white lupin with SQR9 inoculation under low phosphorus conditions (Fig. 6A). In addition, cluster root number of the *LaPIN2* (a auxin transport gene in white lupin) knockdown plants (TRV::*PIN2*) with SQR9 inoculation was significantly reduced compared with wild-type white lupin with SQR9 inoculation under low phosphorus conditions (Fig. 6A). So, the auxin synthesis gene *LaYUC4* in white lupin and auxin-synthesis gene *ysnE* in SQR9 could be the upstream component in regulating SQR9-mediated cluster root formation. Auxin transport gene *LaPIN2* could be the downstream component in regulating SQR9-mediated cluster root formation. As shown in Figure 6B, the pooled-auxins from white lupin *LaYUC4*-producing auxin and bacteria SQR9-producing auxin by its *ysnE* gene were transported by *LaPIN2* gene and then the LaPIN2-transported pooled-auxins promote cluster root formation under low phosphorus conditions (Lines 167, Lines 207-242, Lines 304-311, Lines 519-529, Lines 550-569, Lines 812-822, Lines 844-863).

Question#3: SQR9 mainly increases transcriptional expression of auxin biosynthesis gene YUCs. In stead of auxin transport inhibitor NPA, authors should detect the effects of auxin biosynthesis inhibitor L-kynurenine and auxin precursor L-tryptophan on SQR9-meidated cluster root formation under low phosphorus.

Answer: Thank you for the valuable comments. We added auxin biosynthesis inhibitor L-kynurenine and auxin precursor L-tryptophan experiments to validate the interaction between auxin and cluster root formation. The results showed that L-tryptophan addition positively increased white lupin cluster root formation. However, the L-kynurenine addition inhibited white lupin cluster root formation (Lines 144-155, Lines 262-265, Lines 877-884 and Fig. S2).

Question#4: As shown in Figure 2, SQR9 specifically induces cluster root formation under low phosphorus but not under normal phosphorus conditions. The situations in response to normal phosphorus are important controls for all other studies in Figures 3, 4 and 5. Authors should include the relevant data in the manuscript.

Answer: Many thanks. Yes, we did this as suggested. We re-conducted the relevant experiments under normal phosphorus supply and low phosphorus conditions (Figs. 3A, 4A and Fig. 6A). And, the relevant data have been added in the revised manuscript (Figs. 3B-C, 4B-C and Fig. 6A).

Question#5: In addition to the direct auxin measurements, it's better also to visualize auxin distribution in roots of white lupin using the auxin response reporters, like DR5rev:GFP.

Answer: Thanks for your comments. Yes, we did this as suggested. We used the auxin response reporter DR5rev::GFP plasmid and transferred it into white lupin. The results of auxin measured by high-performance liquid phase showed that the indole-3-acetic acid (IAA, a type of auxin) concentration of roots in white lupin with SQR9 inoculation was significantly increased compared to the uninoculated plants under low phosphorus conditions (Fig. S3A). DR5rev::GFP fluorescence (Figs. S3B, C) also showed that the indole-3-acetic acid concentration of roots in white lupin with SQR9 inoculation was significantly increased. Both the high-performance liquid phase auxin measurement and the auxin response reporter DR5rev::GFP method can

accurately determine the auxin concentration in white lupin root under LP (low phosphorus) with SQR9 inoculation (Lines 157-159, Lines 260-261, Lines 889-897 and Figs. S3A-C).

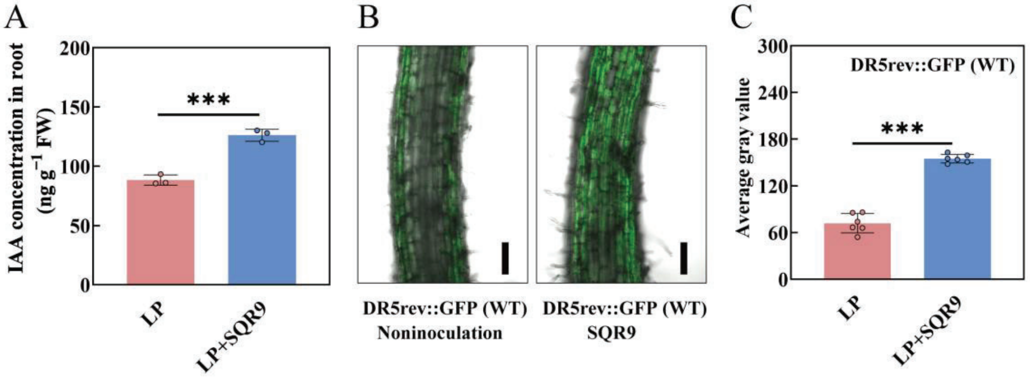

Supplementary Figure 3. The auxin concentration was increased in white lupin root under LP (low phosphorus) with SQR9 inoculation by the high-performance liquid phase measurement or the auxin response reporter DR5rev::GFP method.

Question#6: Figure 5E lacks controls. Author should also quantify cluster root numbers of SQR9- and *ΔysnE* mutant-inoculated plants with empty vector.

Answer: Many thanks for your comments! Yes, we added the controls in the adjusted Fig. 6A. TRV (WT) means the wild-type white lupin with empty vector. The cluster root numbers of TRV (WT) under LP or SQR9 or *Δ ysnE* mutant was almostly same to that of wild-type white lupin without empty vector.

Question#7: Only 4 plants are used to quantify cluster root numbers in the manuscript. To make data more solid, authors should increase the amount of plants for quantifications.

Answer: Many thanks for your constructive comments! To make data more solid, we had increased to 6-8 plants for quantifications in the revised manuscript (Figs 2, 3, 4, 5, 6 and Fig. S2).

Question#8: The manuscript requires some improvement in English language usage.

Answer: Thanks for your comments. We had revised the manuscript as advised. We also asked help from native English editing company to revised english writing problems, please see attached editing certificate as follow (Lines 1-2, Line 32, Line 36, Line 42, Lines 44-51, Line 60, Line 62, Lines 64-66, Lines 68-69, Lines 71-75, Lines 77-80, Lines 82-90, Lines 94-95, Lines 98-101, Lines 103-104, Line 106, Lines 108-109, Line 114, Lines116-129, Lines 131-132, Lines 136-155, Lines157-167, Lines 169-176, Lines 180-184, Lines 189-242, Lines 246-248, Lines 252-253, Lines 259-265, Lines 267-278, Lines 282-294, Lines 299-300, Line 302, Lines 304-311, Lines 328-329, Lines 331-332, Lines 353-355, Lines 358-369, Lines 383-395, Lines 401-402, Line 413, Line 415, Line 417, Lines 419-420, Line 426, Line 428, Lines 433-446, Lines 473-478, Lines 481-491, Lines 495-515, Lines 519-529, Lines 534-546, Lines 550-569, Lines 587-590, Lines 658-659, Lines 671-673, Lines 702-704, Lines 719-721, Lines 741-743, Lines 762-767, Lines 770-780, Lines 785-805, Lines 812-822, Lines 827-839, Lines 844-863, Lines 868-870, Lines 877-884, Lines 889-897, Lines 901-903, Lines 906-911, Lines 914-917).

(Textcheck company, http://www.textcheck.com/certificate/o9tM1b)

Textcheck Certificate

Refnum:	24102116
Title:	Bacillus amyloliquefaciens promotes the cluster root formation of white lupin under low phosphorus through auxins mediation transport
Date:	2024/10/26

We hereby certify that Textcheck has checked and corrected the English in the manuscript named above.

A specialist editor with suitable professional knowledge (M.Sc. or Ph.D./M.D.) reviewed and corrected the English. An English language specialist subsequently checked the paper again. The first language of both editors is English.

Please direct any questions regarding this certificate or the English in the certified paper to: certified@textcheck.com
(Please quote our reference number: '24102116')

Question#9: In additional to quantified data, please at least show one representative image of the cluster roots phenotype.

Answer: Thank you very much. The cluster roots phenotype has been added in Figs.1, 2A, 3A and Fig. 4A of the revised version.

Comments from the Referee#2:

Question#1: Fig. 2B, 3B-D, 4B, and 5D, E should show phenotypic figures, not just statistical results.

Answer: Thank you for your good comments. These phenotypic figures have been added in Figs 2A, 3A, 4A, 5C and Fig. 5F of the revised version.

Question#2: The relative expression of YUC2, YUC3, YUC7, YUC9, and PIN2 in SQR9-inoculation roots was significantly up-regulated according to Fig. 5A, B, why only LaYUC4 knockdown plants TRV::*LaYUC4* was significantly decreased the cluster root number under low phosphorus compared with empty vector control according to Fig. 5D?

Answer: After SQR9 inoculation, the relative expression of *LaYUC*3, *LaYUC*7, *LaYUC*9, *LaYUC4* and *LaPIN2* were up-regulated by 1.95-, 3.54-, 4.64-, 12.23- and 2.83-fold respectively, compared with noninoculation plants under low phosphorus conditions (Figs. 5A, B). However, the relative expression of *LaYUC*2 was decreased by 19.4% (Fig. 5A in the revised version). Among those key auxin genes, the relative expression of *LaYUC4* was the highest, which showed that *LaYUC4* may played a crucial role in SQR9-induced white lupin cluster root formation under low phosphorus conditions. Therefore, we constructed *LaYUC4* silenced lines. *LaYUC4* gene expression and cluster root number of the *LaYUC4* knockdown plants (TRV::*LaYUC4*) were significantly reduced by 72.4% and 38.9%, respectively,

compared with empty vector control plants under low phosphorus conditions (Figs. 5C-E). The *LaYUC4* gene is a representative gene for the YUC genes family. Thus, we only constructed *LaYUC4* silenced lines.

Question#3: YUC4 is an auxin synthesis gene, which increases auxin content. External auxin application promotes the formation of cluster root under low phosphorus conditions according to Fig. 3C. Additionally, the increased auxin content in SQR9 also contributes to the promotion of cluster root formation under low phosphorus conditions, as depicted in Figure 4. However, the involvement of the *LaYUC4* gene from white lupin in the SQR9-mediated promotion of cluster root formation under low phosphorus conditions, as indicated in Figure 5E. how to explain this result?

Answer: Thanks for your comments. The *ysnE* is a key gene for auxin biosynthesis in SQR9. We found that *ysnE*-defective of SQR9 mutant inoculation could not significantly increase cluster root number in white lupin under low phosphorus conditions (Figs. 4A, B and Fig. 6A). Furthermore, cluster root number of the *LaYUC4* (a auxin biosynthesis gene in white lupin) knockdown plants (TRV::*LaYUC4*) with SQR9 inoculation was significantly reduced compared with wild-type white lupin with SQR9 inoculation under low phosphorus conditions (Fig. 6A). In addition, cluster root number of the *LaPIN2* (a auxin transport gene in white lupin) knockdown plants (TRV::*PIN2*) with SQR9 inoculation was significantly reduced compared with wild-type white lupin with SQR9 inoculation under low phosphorus conditions (Fig. 6A).

Firstly, cluster root number of wild-type white lupin with SQR9 inoculation was significantly higher than that of noninoculated wild-type white lupin under low phosphorus conditions. However, cluster root number of wild-type white lupin with *ΔysnE* (The *ΔysnE* showed a 73.7% lower auxin content than SQR9) inoculation was significantly lower than that of wild-type white lupin with SQR9 inoculation under low phosphorus conditions (Fig. 6A and Fig. S5). These results showed that the

SQR9-produced auxin was necessary for SQR9-promoting cluster root formation in white lupin. Secondly, cluster root number of the *LaYUC4* knockdown plants (TRV::*LaYUC4*) with SQR9 inoculation was significantly reduced compared with wild-type white lupin or TRV (WT) with SQR9 inoculation under low phosphorus conditions (Fig. 6A), which showed that the white lupin *LaYUC4*-produced auxin was also necessary for cluster root formation with participanting-SQR9. Thirdly, cluster root number of the *LaPIN2* knockdown plants (TRV::*PIN2*) with SQR9 inoculation was significantly reduced compared with wild-type white lupin with SQR9 inoculation under low phosphorus conditions (Fig. 6A). These results showed that the white lupin auxin polar transport gene *LaPIN2* was essential for cluster root formation with participanting-SQR9 under low phosphorus conditions. Finally, cluster root number of the TRV::*PIN2* with SQR9 inoculation was significantly lower than that of TRV::*LaYUC4* with SQR9 inoculation or wild-type white lupin with SQR9 (*ysnE* gene contributes to SQR9 auxin production) inoculation under low phosphorus conditions. Taken together, we proposed that pooled-auxins from white lupin *LaYUC4*-producing auxin and bacteria SQR9-producing auxin by its *ysnE* gene were transported by *LaPIN2* gene and then the LaPIN2-transported pooled-auxins promote cluster root formation under low phosphorus conditions (Fig. 6B).

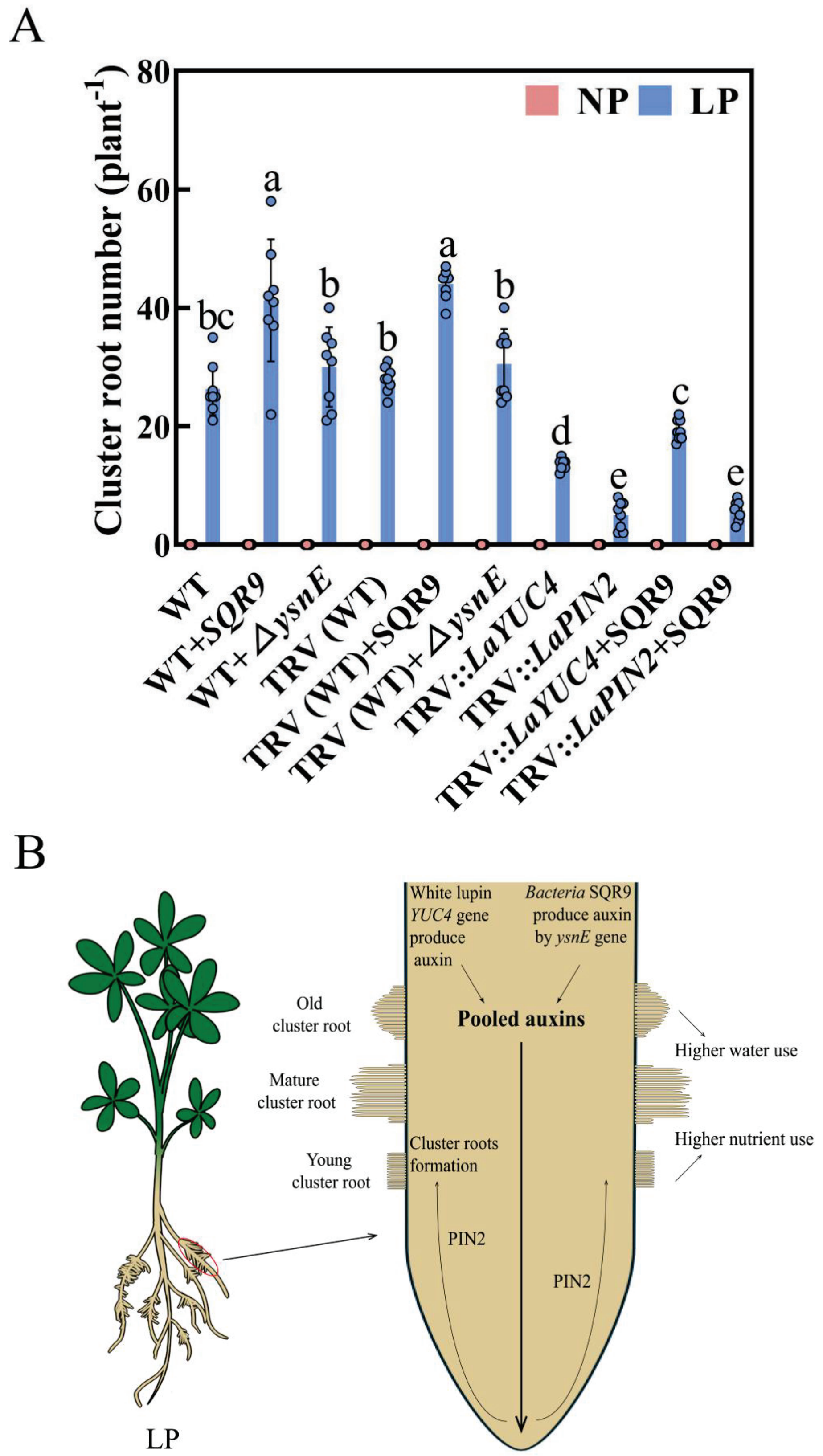

Figure 6. ***Bacillus amyloliquefaciens*** **promotes the cluster root formation of white lupin under low phosphorus through auxins mediation (auxin biosynthesis and**

transport).

Question#4: NPA inhibited the number of cluster root under low phosphorus conditions according to Fig. 3B, indicating that the polar transport of auxin in white lupin plays a crucial role in the formation of cluster root. And under NPA treatment, even the application of SQR9 could not promote the increase of cluster root number under low phosphorus conditions according to Fig. 3D. Therefore, SQR9 produced auxin might be absorbed by plants through polar auxin transport. Authors need to investigate which auxin transport might be involved.

Answer: Integrating the previous directive comments, we proposed that pooled-auxins from white lupin *LaYUC4*-producing auxin and bacteria SQR9-producing auxin by its *ysnE* gene were transported by *LaPIN2* gene. After that, the LaPIN2-transported pooled-auxins promote cluster root formation under low phosphorus conditions (Fig. 6).

Question#5: There is no normal phosphorus treatment control in Figures 3A, 4A, and 4C. And the difference in fresh root weight was not related to low phosphorus conditions according to Fig. 2D.

Answer: Thank you for good comments! Yes, we did this as suggested. We re-conducted the relevant experiments under normal phosphorus supply and low phosphorus conditions (Fig. 2, Fig. 3, Fig. 4 and Fig. 6). According to these results, white lupin did not form cluster roots both in SQR9-inoculated and uninoculated plants under normal phosphorus conditions. Fig. 2D has been adjusted to Fig. 2E in the revised version. Root fresh weight of white lupin with SQR9 inoculation was significantly increased compared to noninoculated plants under normal phosphorus. Root fresh weight of white lupin with SQR9 inoculation was also significantly increased compared to noninoculated plants under low phosphorus conditions (Fig. 2E). In addition, white lupin did not form cluster roots neither SQR9-inoculated nor

noninoculated plants under normal phosphorus conditions. However, cluster root number of white lupin with SQR9 inoculation was significantly higher than that of noninoculated wild-type white lupin under low phosphorus conditions (Fig. 2D). Thus, the increased root fresh weight of white lupin with SQR9 inoculation contributed to cluster root formation.

Question#6: Given the increase in cluster root numbers under low phosphorus conditions, as depicted in Figure 2B, it remains inconclusive whether the rise in IAA content in roots, as shown in Figure 3A, is directly attributable to SQR9.

Answer: To investigate whether the increased root Indole-3-acetic acid content is directly attributable to SQR9, we measured the root Indole-3-acetic acid content with SQR9 inoculation under low phosphorus conditions. Indole-3-acetic acid content of the SQR9-inoculation white lupin was significantly increased by 2.02-fold compared to the noninoculated plants under low phosphorus conditions (Fig. 3D and Figs. S3A-B). The white lupin *LaYUC4* gene produced auxin. Also, *B. amyloliquefaciens* SQR9 produced auxin by its *ysnE* gene. After that, auxins produced by *LaYUC4* and SQR9 were pooled and transported together by the root *LaPIN2* gene, which contributed to cluster root formation (Fig. 6).

Question#7: In line 573-575, SQR9 may not only be expressed in cortical cells according to Fig. 1. Is SQR9 only colonized in the cluster root? How do you explain that? The fluorescence signal of SQR9-GFP could also be detected in other roots under normal and low phosphorus conditions.

Answer: SQR9 could colonize in all roots under normal phosphorus and low phosphorus conditions. Fig. S1 of the revised version was a representative image of SQR9 colonized in the roots of white lupin. This has been revised to "Fluorescence microscopy of white lupin roots showed the presence of *B. amyloliquefaciens* in roots

using confocal microscopy with a superficial view (strains are in green) ” in Fig. S1 caption of revised manuscript (Lines 869-871).

Question#8: According to Figure 2A, SQR9 promoted total root length under normal phosphorus conditions, but inhibited total root length under low phosphorus compared with normal phosphorus conditions. The author should explain this result.

Answer: To make data more solid, we had been increased the amount of plants for quantifications from four plants to eight plants. SQR9 was significantly promoted total root length under normal phosphorus conditions in Fig. 2C of the revised version. The total root length of white lupin with SQR9 inoculation under low phosphorus was lower than that under normal phosphorus conditions, but not significantly (Fig. 2C). This may be due to SQR9-promoted cluster root formation in white lupin under low phosphorus conditions (Fig. 2B). White lupin did not form cluster roots in SQR9-inoculated plants under normal phosphorus conditions (Fig. 2B).

Question#9: In line 154-156, the relative expression of IAA27 and PIN3 in SQR9-inoculation roots were not increased according to Fig. 5A, the description is incorrect.

Answer: Thanks for your comments. The sentence has been changed in the revised version (Lines 198-200).

Question#10: The author should explain the meaning of cluster root in the introduction.

Answer: Many thanks for your good comments! The definition of cluster root has been added in the introduction (Lines 78-80 and Fig.1).

Question#11: In line 81-83, sucrose may contribute to the cluster root formation in white lupin under low phosphorus conditions, the authors could examine whether *Bacillus amyloliquefaciens* SQR9 affected sugar content.

Answer: Thanks for your comments. We determined the sucrose concentration of white lupin roots. There was no significant difference between SQR9-inoculated and uninoculated plants under normal phosphorus or low phosphorus conditions, which indicating that sucrose was not possibley involved in SQR9-induced plant growth of white lupin (Fig. S4). So, we have changed it (Lines 163-166).

Question#12: Why are the statistical differences between Fig. 5A and 5B inconsistent?

Answer: The q-PCR data of Fig. 5A and 5B came from two independent experiments. The data in Fig. 5A and 5B were analyzed with the analysis of variation according to SPSS software (v.24.0), and significant differences were determined by two-sided Student's *t*-test. The *P* value of *LaYUC4* gene in Fig. 5A was 0.00092*,* which was less than 0.001. The difference was statistically marked with 3 stars. However, The *P* value of *LaYUC4* gene in Fig. 5B was 0.00109*,* which was greater than 0.001. The difference was statistically marked with 2 stars.

Question#13: In line 201-204, the conclusion is not accurate.

Answer: Thank you for pointing out the error. The sentence has been changed (Lines 267-269).

Question#14: In line 163, the author forgot to label Fig. 5B.

Answer: We apologize for the lack of a label. The sentence has been changed (Line 189).

Question#15: Why put "or bacterium" at the end of the title?

Answer: The title has been revised to "*Bacillus amyloliquefaciens* promotes the cluster root formation of white lupin under low phosphorus through auxins mediation" (Lines 1-2).

Question#16: In many instances, the results descriptions only describe the experimental results without presenting the corresponding conclusions.

Answer: Thanks for your comments. We added the the corresponding conclusions in the result part as suggested (Lines 131-132, Lines 174-176, Lines 213-214, Lines 217-218, Lines 223-224, Lines 227-228, Lines 232-233, Lines 235-242).

Thanks again to the editor and referees for their careful reviewing.

With Best Wishes,

Yours truly,

Weifeng Xu

Dr. Weifeng Xu, Professor of Plant and Soil in Plant Water-use and Nutrition Regulation and College of JunCao Science and Ecology, Fujian Agriculture and Forestry University, Fuzhou, China, Email: wfxu@fafu.edu.cn.

26-Nov-2024
Prof. Dr. Weifeng Xu
福建农林大学
Jinshan Fuzhou, orcid||||||
China

MSID: PP2024-RA-02780D
MS TITLE: B.amyloliquefaciens promotes the cluster root formation of white lupin under low phosphorus through auxins mediation

Dear Dr. Weifeng Xu:

I am pleased to inform you that your revised manuscript "B.amyloliquefaciens promotes the cluster root formation of white lupin under low phosphorus through auxins mediation" has been accepted for publication in Plant Physiology. Your manuscript will be checked for consistency with journal formatting and image standards by one of our Science Editors, who will contact you shortly. They may have a number of queries and suggestions for you. Please keep in mind that they work to increase the online visibility of your article and its attraction for readers from the widest of backgrounds. You will have the opportunity to review any revisions, and I hope you will work constructively with them to get the most out of your research. You will also have the opportunity to review all changes when you receive the author proofs for your article.

Once validated for publication, your article will be sent to a Science Editor for a final review of your manuscript and source files. After your final files are uploaded, your article will appear in the next available issue and the online Preview version of your article will be posted after your signed Copyright and Checklist forms and final source files are received.

Please be sure to include the following in your manuscript file:
Harvard style citations
List of all author contributions and funding information at the beginning of manuscript after the titles and author list.
List all supplemental materials associated with the article, with titles, at the end of the ms, before the references and acknowledgments section.
Complete Figure Legends for all figures appearing in the article listed before the Literature Cited.

LICENSE INFORMATION Shortly after your paper is sent to production, you will receive an email from our publisher's author support team, **SciPris**, with information on article processing charges, as well as the types of Open Access license you would be able to purchase. The accepted version of your manuscript will not appear on our Advance Articles page until the license is selected and signed, so please look out for an email from SciPris. For more information see the Article Publication Fees section of our author guidelines: https://academic.oup.com/plcell/pages/General-Instructions

Finally, we encourage your submission of artwork for the journal cover. If there is an image or illustration related to your paper that you would like to have considered, please email it in .pdf or .tif format to clowe@aspb.org. An image title, brief, 2-3 sentence description of the image, and the name of the person credited for the image are also encouraged. If your image is selected to appear on the online cover of the journal, you will be contacted with the scheduled issue date and further information.

Thank you and congratulations on your Plant Physiology paper!

Sincerely,
Editor, Plant Physiology

=====
FIRST AUTHOR PROFILES
=====

We recognize the hard work that goes into being First Author of a Plant Physiology paper by publishing a "First Author Feature" for each First Author alongside your paper.

If the first author(s) would like to submit information for a feature on Facebook and Twitter, fill out the form by clicking on the link below:

Plant Physiology First Author Feature Form: https://forms.office.com/r/ZBCGZfhm8F
If you cannot access the form through the link above, please contact Rachel at rbelsky@aspb.org.

To upload an image of yourself to accompany your profile, please put your photo in the correct journal folder at the link below. You must label your image: Author First Name Last Name Article Title.

First Author Profile Image Dropbox: https://www.dropbox.com/scl/fo/xz029p7qjr2jml2rp2kmq/h?dl=0&rlkey=6fkxcaqmxx35ap152b2osy1fz

=====
IMPORTANT REMINDER: PEER REVIEW REPORTS
=====

If you opted to publish a peer review report along with your article during the original submission process, it will be prepared by the editorial staff and publicly posted with your manuscript, inside the zip file that contains any other supplemental material. As a reminder, the peer review report is a public record of all comments from editors and reviewers, as well as your prior responses, as you received them in the decision letters for each draft of your manuscript. If you agreed to publish this report and have changed your mind, or are not sure if you selected this option, please contact the editorial office as soon as possible before signing the license agreement from our publisher.

-- Reviewer comments:
Reviewer #1 (Comments for the Author):

My concerns have been addressed in the revised manuscript.

Reviewer #2 (Comments for the Author):

I am happy with the revisions, and have no further comments.
